# Alzheimer’s disease: insights into pathology, molecular mechanisms, and therapy

**DOI:** 10.1093/procel/pwae026

**Published:** 2024-05-11

**Authors:** Qiuyang Zheng, Xin Wang

**Affiliations:** Shenzhen Research Institute of Xiamen University, Shenzhen 518057, China; State Key Laboratory of Cellular Stress Biology, Fujian Provincial Key Laboratory of Neurodegenerative Disease and Aging Research, Institute of Neuroscience, Department of Neurology, the First Affiliated Hospital of Xiamen University, School of Medicine, Xiamen University, Xiamen 361005, China; Shenzhen Research Institute of Xiamen University, Shenzhen 518057, China; State Key Laboratory of Cellular Stress Biology, Fujian Provincial Key Laboratory of Neurodegenerative Disease and Aging Research, Institute of Neuroscience, Department of Neurology, the First Affiliated Hospital of Xiamen University, School of Medicine, Xiamen University, Xiamen 361005, China

**Keywords:** Alzheimer’s disease, pathophysiology, risk factors, biomarkers, prevention

## Abstract

Alzheimer’s disease (AD), the leading cause of dementia, is characterized by the accumulation of amyloid plaques and neurofibrillary tangles in the brain. This condition casts a significant shadow on global health due to its complex and multifactorial nature. In addition to genetic predispositions, the development of AD is influenced by a myriad of risk factors, including aging, systemic inflammation, chronic health conditions, lifestyle, and environmental exposures. Recent advancements in understanding the complex pathophysiology of AD are paving the way for enhanced diagnostic techniques, improved risk assessment, and potentially effective prevention strategies. These discoveries are crucial in the quest to unravel the complexities of AD, offering a beacon of hope for improved management and treatment options for the millions affected by this debilitating disease.

## Introduction

Alzheimer’s disease (AD), the predominant type of neurodegenerative disorder, is characterized by the extracellular buildup of β-amyloid (Aβ) plaques and the intracellular aggregation of neurofibrillary tangles (NFTs), which are composed of hyperphosphorylated tau. Additionally, AD is characterized by the loss of synapses and neurons, as well as gliosis (Long and Holtzman, 2019). Since its initial identification by Alois Alzheimer in the early 20th century, AD has emerged as a significant healthcare challenge, and no cure is currently available (Alzheimer et al., 1995).

## Clinical signs and symptoms

AD presents a spectrum of clinical symptoms, initially manifesting as early amnestic cognitive impairment and difficulties with short-term memory. As AD progresses, affected individuals may experience impairments in complex attention, expressive speech, visuospatial processing, and executive functions (McKhann et al., 2011; Tarawneh and Holtzman, 2012). Neuropsychiatric symptoms frequently accompany cognitive deficits in AD, particularly in earlier stages, when anxiety, depression, and apathy are prevalent. With disease progression, patients may develop additional symptoms, including delusions, hallucinations, agitation/aggression, and irritability/lability (D’Onofrio et al., 2012).

Historically, AD diagnosis was limited to the dementia stage, characterized by significant, progressive cognitive impairment across various domains or neurobehavioral symptoms severe enough to markedly impair daily functioning (Scheltens et al., 2021). With advancements in biomarker research, Clifford Jack and colleagues redefined AD diagnosis from a purely clinical syndrome to a biological framework based on biomarkers. These biomarkers are categorized into Aβ deposition (A), pathological tau (T), and neurodegeneration (N) biomarkers. The diagnosis of AD, according to this research framework, depends on the presence of Aβ and phosphorylated tau (Jack et al., 2018). The ATN framework underscores the critical roles of Aβ and tau in diagnosing AD, distinctly identifying AD as a unique neurodegenerative disease among various dementia-causing disorders.

In 2010, Clifford Jack et al. introduced a hypothetical model of dynamic biomarkers for AD, spanning the cognitive continuum from health to dementia (Jack et al., 2010). They segmented the clinical disease stages of AD into three phases: the presymptomatic phase, the prodromal phase (often referred to as mild cognitive impairment (MCI)), and the dementia phase. AD pathology may initially be asymptomatic, manifesting through a stage of MCI before progressing to overt dementia. The onset of Aβ pathology in individuals predisposed to AD occurs approximately 15–20 years before the anticipated onset of progressive cognitive decline (Fleisher et al., 2012; Reiman et al., 2012). Elevated cerebrospinal fluid (CSF) tau levels indicate neuronal injury and correlate with disease severity. ^18^fluorodeoxyglucose (^18^FDG)-positron emission tomography (PET) is a reliable marker of synaptic dysfunction associated with neurodegeneration in AD patients. Structural magnetic resonance imaging (MRI) quantifies cerebral atrophy as synapses and neurons deteriorate, which is closely correlated with the severity of clinical impairment, even in the disease’s advanced stages, and aligns with Braak staging and tau tangle pathology observed postmortem (Jack et al., 2010).

## Epidemiology

AD represents 60%–80% of dementia cases (Alzheimer's Association., 2023). In 2019, the global number of individuals with dementia was approximately 57.4 million. From 2000 to 2019, the number of deaths attributable to AD increased by more than 145%, indicating that AD is the sixth leading cause of death in the United States (Alzheimer's Association., 2023).

Approximately 1% of AD cases fall under the category of early-onset autosomal dominant AD, with the remaining 99% being late-onset sporadic AD. Autosomal dominant AD typically manifests before age 65, with many individuals experiencing symptoms in their 40s and 50s (Bateman et al., 2011; Ryman et al., 2014).

Genetic predispositions significantly influence the pathophysiological mechanisms of AD, accounting for an estimated 58%–79% of cases (Gatz et al., 2006). Rare mutations in *APP*, *PSEN1,* and *PSEN2* are linked to autosomal dominant AD (Bertram et al., 2010). The apolipoprotein E (APOE) gene is a major genetic risk factor for sporadic AD (Corder et al., 1993; Peacock et al., 1993). The prevalence of *APOEε4* was 66% among patients with AD-type dementia and 64% among those with MCI (Mattsson et al., 2018). Possession of one *APOEε4* allele amplifies the risk 3–4 times, whereas two alleles heighten the risk by 9–15 times (Farrer et al., 1997; Genin et al., 2011; Neu et al., 2017). Whole-genome sequencing (WGS) and genome-wide association studies (GWAS) have revealed additional genetic loci associated with late-onset AD risk, including *TREM2*, *BIN1*, *CLU*, *ABCA7*, *CR1*, *PICALM*, *MS4A6A*, *CD33*, *MS4A4E*, *CD2AP*, *EPHA1*, and *EXOC3L2*/*BLOC1S3*/*MARK4*, among others (Guerreiro et al., 2013; Harold et al., 2009; Hollingworth et al., 2011; Jonsson et al., 2013; Lambert et al., 2009; Naj et al., 2011; Seshadri et al., 2010). A meta-analysis further identified several susceptibility loci for late-onset AD, including *HLA*-*DRB5*-*HLA*-*DRB1*, *PTK2B*, *SORL1*, *SLC24A4*-*RIN3*, *DSG2*, *INPP5D*, *MEF2C*, *NME8*, *ZCWPW1*, *CELF1*, *FERMT2*, and *CASS4* (Lambert et al., 2013).

Age remains the most significant risk factor for developing AD. Approximately 18.1% of individuals aged ≥ 65 years are affected by AD, a figure that rises to 33.2% among those aged ≥ 85 years (Alzheimer’s Association., 2023). Additionally, in the United States, 21.1% of women and 11.6% of men over 65 years old are affected by AD (Alzheimer's Association., 2023).

The Lancet Commission on Dementia Prevention has identified 12 modifiable risk factors that collectively account for approximately 40% of dementia worldwide. These factors include lower education levels, hypertension, hearing impairment, smoking, obesity, depression, physical inactivity, diabetes, and limited social interaction (Livingston et al., 2020).

## Pathophysiology

### Aβ

The accumulation of Aβ aggregates in the brain is a fundamental cause of AD (Long and Holtzman, 2019). Aβ was initially identified as the main component of cerebrovascular amyloid (Glenner and Wong, 1984) and was later identified as the central component of cerebral amyloid plaques in both AD patients and aged individuals with Down syndrome (DS) (Masters et al., 1985).

#### β-Amyloid precursor protein (APP) processing

The gene encoding APP is located on human chromosome 21. APP is a type-I transmembrane protein with an extracellular amino (N)-terminus and an intracellular carboxyl (C)-terminus oriented intracellularly (Dyrks et al., 1988; Kang et al., 1987). It exists in three primary isoforms due to alternative splicing: APP695, APP751, and APP770. The APP695 isoform, comprising 695 amino acids, is primarily found in neurons. In contrast, the APP751 and APP770 isoforms, containing 751 and 770 amino acids, respectively, are ubiquitously expressed across various tissues (Rohan de Silva et al., 1997). According to the AlzForum Mutations database, researchers have identified 20 pathogenic mutations within the *APP* gene in individuals diagnosed with AD. The APP-A673T variant (Icelandic mutation) is the sole variant known to confer protection against Aβ generation (Peacock et al., 1993).

**Figure 1.**
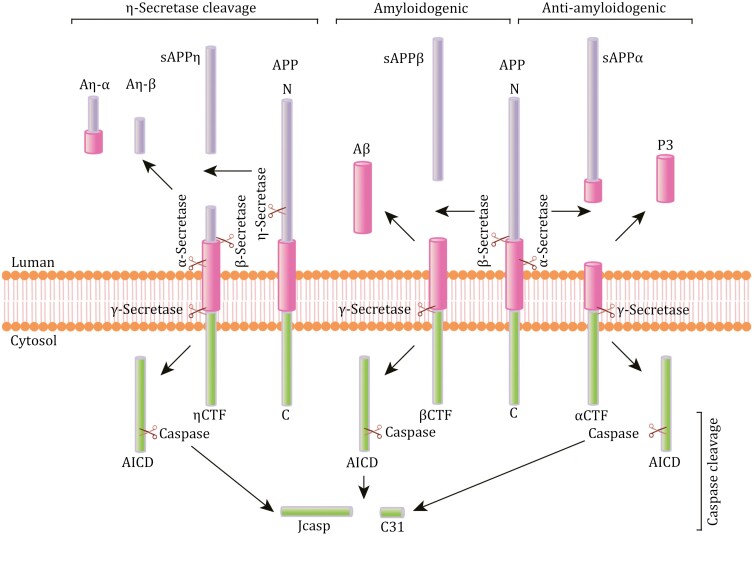
Illustration of APP processing pathways. In the amyloidogenic pathway, APP undergoes cleavage by β- and γ-secretase, leading to the production of Aβ. Alternatively, the anti-amyloidogenic pathway results in the generation of neuroprotective sAPPα instead of Aβ. The processing of APP is also influenced by the action of η-secretase and caspases.

APP undergoes two main processing pathways: the amyloidogenic pathway, which leads to the production of neurotoxic Aβ, and the anti-amyloidogenic pathway, which inhibits Aβ generation (Fig. 1).

In the amyloidogenic pathway, APP is sequentially proteolytically cleaved by β- and γ-secretase, resulting in the formation of Aβ peptides (Zhang et al., 2011). The initial cleavage by β-secretase occurs at the N-terminus of Aβ, releasing the soluble ectodomain of APP (sAPPβ) and the membrane-bound APP carboxyl-terminal fragment (βCTF or C99). BACE1 has been identified as the primary β-secretase, functioning optimally in acidic conditions found in the Golgi and endosomal organelles (Huse et al., 2000, 2002; Hussain et al., 2000; Lin et al., 2000; Sinha et al., 1999; Vassar et al., 1999; Walter et al., 2001; Yan et al., 1999). The γ-secretase complex further cleaves C99, releasing various Aβ peptides and the APP intracellular domain (AICD) (Bibl et al., 2006). Among these peptides, Aβ_40_ and Aβ_42_ are predominant in the AD brain, with Aβ_42_ being prone to aggregation and becoming more neurotoxic due to its hydrophobic C-terminus (Roher et al., 1993). The γ-secretase complex consists of four protein subunits, namely, presenilin (PS1 or PS2), presenilin enhancer 2 (PEN2), APH2, and nicastrin (Kimberly et al., 2003; Takasugi et al., 2003), with mutations in *PSEN1* or *PSEN2* implicated in most cases of autosomal dominant AD (Levy-Lahad et al., 1995; Sherrington et al., 1995).

In the anti-amyloidogenic pathway, APP undergoes sequential cleavage by α- and γ-secretase. α-Secretase cuts APP within the Aβ domain, producing a truncated APP CTF (αCTF or C83) and a soluble ectodomain (sAPPα) (Esch et al., 1990; Sisodia et al., 1990). sAPPα is crucial for neuronal plasticity and survival, offering protection to hippocampal neurons from excitotoxicity (Furukawa et al., 1996). Moreover, sAPPα alone has been demonstrated to mitigate behavioral abnormalities and synaptic deficits in APP-knockout mice (Ring et al., 2007). α-Secretase activity is attributed to three members of the ADAM (a disintegrin and metalloproteinase) family: ADAM9, ADAM10, and ADAM17, also known as tumor necrosis factor-α converting enzymes (TACE) (Zhang et al., 2011). Subsequently, γ-secretase further cleaves C83, releasing the truncated Aβ peptides P3 and AICD (Haass et al., 1993; Sastre et al., 2001).

Beyond the classical APP processing pathway, APP can be cleaved by the membrane-bound matrix metalloproteinase η-secretase, such as MT5-MMP. This cleavage generates soluble truncated ectodomains of APP (sAPPη) and ηCTF. Subsequently, ηCTF undergoes cleavage by ADAM10 and BACE1, yielding the peptides Aη-α and Aη-β (Fig. 1), and ηCTF is enriched in dystrophic neurites. Like Aβ, Aη-α exhibits neurotoxic effects (Willem et al., 2015).

Additionally, APP is subject to cleavage by caspases, predominantly caspase-3, at the D664 residue within its C-terminus (based on APP695 numbering), resulting in a C-terminal 31-amino acid peptide (C31). γ-Secretase cleavage also produces the Jcasp fragment, which spans the region between the γ- and caspase-cleavage sites (amino acids 649–664; Fig. 1) (Gervais et al., 1999; Park et al., 2009). Both C31 and Jcasp are associated with neurotoxicity (Lu et al., 2003). Importantly, blocking caspase cleavage in a human APP transgenic mouse model with the D664A mutation led to reduced synaptic loss, astrogliosis, and cognitive deficits despite the presence of abundant Aβ plaques in the brain (Galvan et al., 2006).

#### Aβ seeding and spreading

Due to their hydrophobic amino acids, Aβ monomers tend to aggregate and form oligomers. These oligomers range from low-molecular-weight species such as dimers and trimers to intermediate-sized nonamers, dodecamers, and high-molecular-weight oligomers (protofibrils). The interaction between the hydrophobic amino acids in protofibrils or oligomers within amyloid plaques leads to the formation of fibrils. These fibrils then stack together, resulting in plaque formation (Yang et al., 2017).

Recent advancements in cryo-electron microscopy (cryo-EM) have unveiled the structures of Aβ_42_ filaments in the human brain, identifying two distinct filament types characterized by their S-shaped protofilament folds. Type I filaments, which are mainly found in sporadic AD patients, consist of two identical S-shaped intertwined protofilaments. Their structure includes five β-strands and two hydrophobic clusters. Type II filaments, observed in familial AD patients, have smaller protofilament interfaces stabilized by electrostatic interactions. Both filament types exhibit left-handed twists, differing from the Aβ structure in cerebral amyloid angiopathy (CAA) in AD patients. Notably, Aβ_42_ deposits in *App*^*NL-G-F*^-knock-in mice predominantly consist of Type II fibrils (Yang et al., 2022).

Aβ fibrillization can be initiated in a prion-like manner through the formation of misfolded β-sheet-containing Aβ seeds, which act as templates for larger amyloid aggregates (Walker and Jucker, 2015). Experiments involving the intracerebral injection of AD patient-derived brain extracts into marmosets have led to amyloid plaque formation, dystrophic neurites, and cerebral amyloid angiopathy without NFTs (Baker et al., 1993). Similarly, injections of Aβ-containing brain extracts into human APP transgenic mice have been shown to induce cerebral β-amyloidosis and associated pathologies (Eisele et al., 2009, 2010; Fritschi et al., 2014; Meyer-Luehmann et al., 2006). There have also been reports of human-to-human transmission of iatrogenic Aβ in young adults treated with cadaver-derived pituitary growth hormone contaminated with both Creutzfeldt–Jakob disease (CJD) prions and Aβ seeds (Purro et al., 2018).

Several cofactors, including metal ions (Abelein, 2023), glycosaminoglycans (Iannuzzi et al., 2015), APOE (Garai et al., 2014; Hashimoto et al., 2012), α-synuclein (Chia et al., 2017), and β2-microglobulin (B2M) (Zhao et al., 2023), have been implicated in facilitating Aβ aggregation. Notably, B2M, a component of the major histocompatibility complex class I (MHC-I), plays a crucial role in antigen presentation to cytotoxic T lymphocytes and is vital for adaptive immune responses (Cresswell et al., 2005). Recent studies have characterized novel amyloid-like protein aggregates composed of B2M and Aβ in the AD brain. Elevated levels of B2M in the brains of individuals with AD are crucial for Aβ aggregation and neurotoxicity, whereas depletion of B2M reduces amyloid spreading and completely neutralizes the neurotoxicity of Aβ. In contrast to Aβ, peripheral B2M can cross the blood‒brain barrier (BBB) and enter the brain parenchyma (Zhao et al., 2023). Antibody-mediated depletion of peripheral B2M effectively eliminates cognitive deficits in AD and DS mouse models (Gao et al., 2023; Zhao et al., 2023). This research suggests targeting peripheral B2M as a potential strategy for treating cognitive impairment in individuals with AD and DS, potentially overcoming the challenge of drug delivery across the BBB that is commonly encountered in the treatment of central nervous system (CNS) diseases.

The spread of Aβ in AD shares similarities with prion spreading, potentially propagating through neuron-to-neuron transmission (Domert et al., 2014; Nath et al., 2012; Song et al., 2014). Furthermore, the propagation of Aβ pathology, triggered by seeding, may follow neuroanatomical routes aligned with the limbic connectome (Ye et al., 2015). Analysis of florbetapir (^18^F-AV45) PET data revealed the distribution pattern of Aβ pathology. Typically, the deposition of Aβ plaques begins in the temporobasal and frontomedial areas and then gradually extends to encompass the broader associative neocortex, primary sensory-motor regions, and the medial temporal lobe, ultimately reaching the striatum (Grothe et al., 2017a).

#### Amyloid cascade hypothesis

Building on multiple sources of evidence, Hardy and Higgins introduced the amyloid cascade hypothesis in 1992 (Hardy and Higgins, 1992). This hypothesis posits that the increase in the generation and aggregation of Aβ in the brain is the initial step in AD pathogenesis, leading to NFT formation, neuronal loss, and ultimately, cognitive decline.

Individuals with DS who have a third copy of chromosome 21 carry an extra copy of the *APP* gene. This genetic duplication results in increased Aβ deposition and AD-like neuropathology. Additionally, missense mutations in the *APP* gene, identified in patients with autosomal dominant AD, further contribute to AD pathology. Notably, the APP-A673T variant has been linked to a decreased risk of developing AD, providing strong support for the amyloid cascade hypothesis (Tcw and Goate, 2017). Recent clinical trials of monoclonal antibodies targeting Aβ, including lecanemab, donanemab and aducanumab, have shown promising results in slowing the progression of early-stage AD (Budd Haeberlein et al., 2022; Sims et al., 2023; van Dyck et al., 2023). These results offer significant clinical evidence for the crucial role of Aβ in AD pathogenesis and further validate the amyloid cascade hypothesis.

However, the simplicity of this neuron-centric, linear model has been questioned based on clinical observations, challenging the direct causal relationship between Aβ and dementia (De Strooper and Karran, 2016). The severity of cognitive decline has been shown to correlate more closely with the presence of NFTs than with the presence of Aβ plaques (Nelson et al., 2012). Furthermore, regional brain hypometabolism appears to be unrelated to the burden of regional Aβ plaques (Altmann et al., 2015). These insights suggest that while Aβ accumulation may initiate AD progression, other downstream processes, such as neuroinflammation and tau pathology, might be the main drivers of neurodegeneration (Long and Holtzman, 2019).

The ongoing debate surrounding the relative importance of Aβ and tau in AD pathogenesis reflects the complexity of AD and the need for a deeper understanding of the interplay between Aβ and tau in the disease process. It is possible that Aβ and tau each play critical, yet distinct, roles in AD pathogenesis, with Aβ initiating the cascade and tau driving subsequent neurodegeneration. However, the specific mechanisms through which Aβ and tau interact and contribute to the development and progression of AD remain unclear. Further research is required to clarify the exact roles of Aβ and tau in AD pathogenesis. This may include developing new imaging techniques and biomarkers to better monitor disease progression, as well as identifying novel therapeutic targets that address both Aβ and tau pathology.

### Tau

Tau, the primary component of NFTs, plays a crucial role in regulating microtubule stability and intracellular trafficking under normal conditions as a microtubule-associated protein (Vershinin et al., 2007). However, impaired tau function can lead to various neurological issues. For instance, tau deficiency has been shown to disrupt the trafficking of APP, also an iron export protein, to the neuronal surface, resulting in toxic iron retention, cognitive deficits, and parkinsonism-like symptoms in mice (Lei et al., 2012). Tau deficiency protects young mice from ischemic stroke by preventing ferroptosis, and this effect diminishes in older tau knockout mice due to accelerated age-dependent brain iron accumulation (Tuo et al., 2017). Furthermore, additional research indicates that tau may also affect axonal elongation and maturation, synaptic plasticity, and neuronal excitability (Wang and Mandelkow, 2016). The diverse roles of tau highlight its multifaceted nature and its significance in maintaining neuronal homeostasis and overall brain function. Given the involvement of tau in a variety of cellular functions, from microtubule dynamics to synaptic activity, further research into its impact on neuronal processes is crucial.

The regional progression of brain atrophy in AD strongly correlates with tau accumulation, more so than with Aβ deposition (Giannakopoulos et al., 2003). In addition to AD, NFTs are also found in the brains of older individuals who may show no cognitive impairment or only amnestic changes, even in the absence of amyloid plaques. These instances are classified as “primary age-related tauopathy” (PART) (Crary et al., 2014). Moreover, tau pathology appears in other tauopathies, traumatic brain injury (TBI), and stroke (Congdon and Sigurdsson, 2018). Mutations in the tau gene *MAPT*, located on chromosome 17, are linked to several tauopathies, including progressive supranuclear palsy (PSP), corticobasal degeneration (CBD), Pick disease (PiD), and frontotemporal dementia (FTD) (Congdon et al., 2023).

Alternative splicing of the *MAPT* gene, particularly involving exons 2 and 3 at the N-terminus (N) and exon 10 within the microtubule-binding repeat domain (MBRD), leads to six distinct isoforms in the adult human brain: 0N3R, 0N4R, 1N3R, 1N4R, 2N3R, and 2N4R (Goedert et al., 1989). Normally, the brain maintains equal levels of 4R- and 3R-tau isoforms, AD typically presents with a combination of both 4R- and 3R-tau, CBD and PSP show aggregates of 4R-tau only, and PiD features 3R-tau aggregates. In cases of FTD associated with tau pathology, patients often exhibit aggregates of either exclusively 4R- or 3R-tau (Kyalu Ngoie Zola et al., 2023; Spillantini and Goedert, 1998).

In AD patients, the levels of soluble tau are lower, yet there are approximately 100 times more insoluble tau than in controls. Soluble tau tends to be full-length, incorporating both its N-terminal domain and all four MBRDs (2N4Rs). Conversely, the insoluble fraction often lacks N-terminal domains and is predominantly composed of the 0N4R isoform (Wesseling et al., 2020).

#### Posttranslational modification of Tau

**Figure 2.**
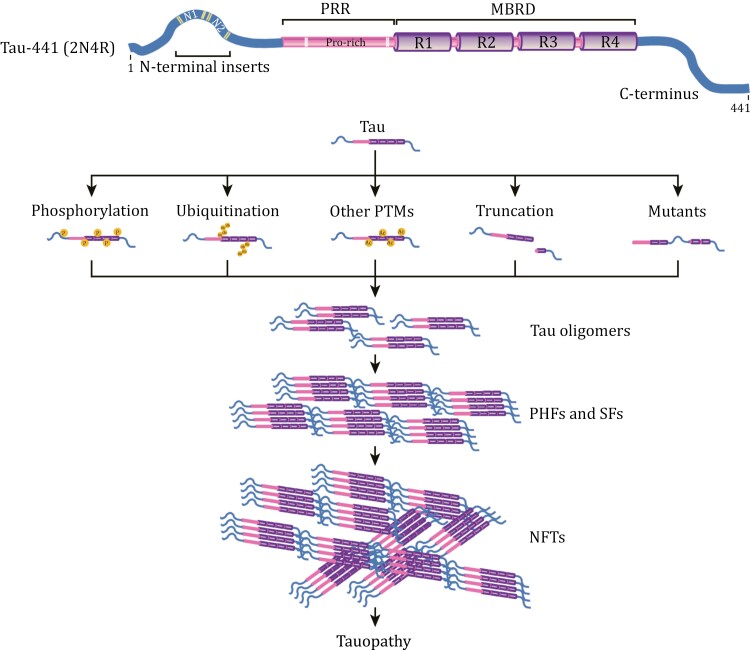
Tau protein and the formation of neurofibrillary tangles. The diagram illustrates the structural features of the 2N4R tau protein. In the adult human brain, six isoforms of tau are produced through alternative splicing of the *MAPT* gene, with variations in the inclusion of exons at the N-terminus and exon 10 in the MBRD. In addition to genetic mutations, PTMs such as phosphorylation, ubiquitination, acetylation, and C-terminal truncation initiate the accumulation of tau into oligomers and filaments, including paired helical filaments (PHFs) and straight filaments (SFs). This accumulation culminates in the formation of NFTs, which are toxic and play a crucial role in the pathogenesis of tauopathies, thereby contributing to their detrimental impacts.

Prior to forming NFTs, the tau protein undergoes an intricate array of posttranslational modifications (PTMs), including phosphorylation (P), ubiquitination (Ub), SUMOylation, acetylation (Ac), methylation, glycosylation and truncation (Fig. 2). Through comprehensive qualitative and quantitative analysis of tau proteoforms from postmortem human brains involving 49 AD patients and 42 controls, researchers identified 95 unique PTMs across 88 amino acid residues, encompassing 55 phosphorylation, 17 ubiquitination, 19 acetylation, and four methylation sites. Additionally, clusters with higher Braak stages displayed increased levels of tau and Aβ as well as elevated tau PTMs. Phosphorylation was most common in the proline-rich region (PRR) and the C-terminus, while ubiquitination and acetylation were mainly localized to the MBRD, with certain residues exhibiting both modifications. In AD patients at Braak stages V-VI, significant PTMs, such as phosphorylation at S262 and S263; acetylation at K311, K353, and K369; and ubiquitination at K259, K267, K311, and K317, are associated with increased tau and amyloid burdens (Wesseling et al., 2020). Furthermore, specific PTMs in soluble tau—Ub-K369 and Ub-K343 for 4R-tau and Ac-K311 and P-S184 + P-S185 for 3R-tau—were identified as key discriminators between 4R- and 3R-tauopathies (Kyalu Ngoie Zola et al., 2023). Additionally, the levels of free UFM1, a type I ubiquitin-like protein, were found to be reduced in the brains of patients with AD or PSP. Knockdown of genes associated with the UFMylation process, including *UBA5* and *UFM1*, reduces tau inclusion formation in human neurons derived from induced pluripotent stem cells (iPSCs) harboring the Tau-P301S mutation. Furthermore, depletion of the UFM1-activating enzyme UBA5 has been shown to ameliorate tau propagation in the PS19 mouse model of tauopathy (Parra Bravo et al., 2024). These findings suggest that the UFMylation pathway plays a role in the pathogenesis of both AD and PSP. Therefore, targeting the UFMylation process could represent a potential therapeutic strategy for these neurodegenerative disorders.

##### Phosphorylation

Tau hyperphosphorylation is a key pathological event in the development of tauopathies. Researchers have identified 86 phosphorylation events on tau, 55 of which are specifically linked to pathologically insoluble tau formation (Wesseling et al., 2020). Hyperphosphorylation of tau, an early pathological marker, impairs its microtubule-stabilizing function, leading to its mislocalization to the somatodendritic compartment (Congdon et al., 2023). Certain phosphorylation sites, such as S199, S202, S205, T231, and S262, are pivotal during the pre-tangle phase of the disease (Augustinack et al., 2002; Luna-Munoz et al., 2007). The emergence of S422 phosphorylation, which correlates with an increase in somatic tau protein (Hanger and Wray, 2010), appears to prevent cleavage at D421, a process occurring before this phosphorylation event. Moreover, phosphorylation at S396 becomes increasingly prominent as AD progresses (Kimura et al., 1996).

Phosphorylation at residues T217, T231, S262, and S396 is known to promote tau aggregation (Guo et al., 2006; Helboe et al., 2022; Kadavath et al., 2018; Savastano et al., 2021). In contrast, phosphorylation at residues T212, S237, and S404 is associated with a reduction in tau aggregation (Povellato et al., 2014). Interestingly, while no unique phosphorylation site on soluble tau has been identified as exclusively indicative of AD, the presence of phosphorylation at T217, T231, and S396 within the soluble tau fraction serves as a potential biomarker to distinguish AD patients from non-AD individuals (Kyalu Ngoie Zola et al., 2023).

Tau phosphorylation is modulated by three main groups of kinases: proline-directed serine-threonine protein kinases, non-proline-directed serine-threonine protein kinases, and tyrosine protein kinases (Guo et al., 2017). Key kinases, such as glycogen synthase kinase 3β (GSK3β), c-JUN N-terminal kinase (JNK), and cyclin-dependent kinase 5 (CDK5), are upregulated in the AD brain (Shukla et al., 2012; Tell and Hilgeroth, 2013; Yarza et al., 2015). In contrast, the activity of protein phosphatase 1 (PP1) and PP2A, which are responsible for dephosphorylating tau, is reduced in postmortem AD brains (Gong et al., 1993; Sontag et al., 2004; Vogelsberg-Ragaglia et al., 2001).

##### Ubiquitination

The accumulation of ubiquitinated proteins within NFTs and amyloid plaques is a hallmark of AD pathology, highlighting the critical role of ubiquitination in the disease process (Mori et al., 1987; Perry et al., 1987). Seventeen out of 40 lysine residues have been pinpointed as ubiquitination sites in the human tau protein (Wesseling et al., 2020). Ubiquitination at residues K311, K317, K321, or K369 reduces kinetic barriers, promoting the formation of tau filaments (Wesseling et al., 2020). The PTMs (Ub-K311, Ub-K317, and Ub-K267 + P-S262) in soluble tau from postmortem human brains have been identified as AD-specific, distinguishing them from other tauopathies, such as CBD, FTD, and PiD (Kyalu Ngoie Zola et al., 2023).

Ubiquitination of tau, facilitated by E3 ligases such as the C-terminus of Hsc70-interacting protein (CHIP), TNF receptor-associated factor 6 (TRAF6) and axotrophin/MARCH7, plays a critical role in tau dynamics (Babu et al., 2005; Flach et al., 2014; Petrucelli et al., 2004). CHIP targets the MBRD of tau, promoting K48- and K63-linked ubiquitination, which leads to increased tau aggregation. Moreover, Hsp70 enhances tau turnover, reducing the levels of both insoluble and hyperphosphorylated tau (Petrucelli et al., 2004), indicating the crucial role of the Hsp70/CHIP chaperone system in managing tau pathology. The role of TRAF6 in mediating K63 polyubiquitination points toward its involvement in the ubiquitin‒proteasome system’s tau degradation pathway (Babu et al., 2005). On the other hand, the effect of axotrophin/MARCH7 on tau monoubiquitylation reduces the microtubule-binding capacity of tau (Flach et al., 2014).

Further research into the role of ubiquitin-specific peptidases, such as X-linked ubiquitin-specific peptidase 11 (USP11), revealed its increase in the AD brain. The ability of USP11 to deubiquitinate tau at K281 leads to enhanced tau acetylation and subsequent pathological aggregation in PS19 mice (Yan et al., 2022). Conversely, the ubiquitin thioesterase Otub1 has been identified as a tau deubiquitinating enzyme that disrupts K48-linked tau deubiquitylation, impairing tau degradation and promoting aggregation (Wang et al., 2017a).

##### SUMOylation

SUMOylation, the covalent attachment of small ubiquitin-related modifier (SUMO) proteins, is another reversible PTM that influences tau function and pathology (Geiss-Friedlander and Melchior, 2007). Tripartite motif 11 (TRIM11), a SUMO E3 ligase for tau, is downregulated in the AD brain. TRIM11 facilitates SUMOylation and proteasomal degradation of mutant tau. Moreover, TRIM11 enhances tau solubility by acting both as a chaperone to prevent its misfolding and as a disaggregated to break down existing tau fibrils (Zhang et al., 2023). Additionally, SUMOylation at the K340 residue of tau facilitates its phosphorylation while simultaneously blocking ubiquitination-mediated tau degradation (Luo et al., 2014).

##### Acetylation

Acetylation is another critical PTM affecting tau, with 23 lysines and two cysteines identified as potential acetylation sites across the tau sequence (Min et al., 2010; Prifti et al., 2021). Acetylation at K274, K280, and K281 disrupts the physiological functions of tau, impairing its interaction with microtubules and leading to its mislocalization and pathological aggregation, which contributes to synaptic deficits in tauopathies (Cohen et al., 2011; Sohn et al., 2016; Tracy et al., 2016). Moreover, acetylation at K163, K174, and K180 has been shown to reduce tau turnover by inhibiting its ubiquitination, highlighting the competitive nature between acetylation and ubiquitination in regulating tau degradation (Min et al., 2010).

Tau acetylation is facilitated by the histone acetyltransferase p300 (Min et al., 2010) and cAMP-response element-binding protein (CREB)-binding protein (CBP) (Kamah et al., 2014). Additionally, autoacetylation at residues C291 and C322 within the MBRD contributed to the initiation of tau aggregation (Prifti et al., 2021). Conversely, deacetylation, orchestrated by sirtuin 1 (Min et al., 2010) and histone deacetylase 6 (HDAC6), plays divergent roles in relation to tau pathology. Sirtuin1 acts protectively, reducing tau aggregation, whereas HDAC6 is associated with increased tau pathology propagation (Carlomagno et al., 2017). Furthermore, the deacetylation of residues within the KXGS motif by HDAC6 enhances tau phosphorylation and aggregation, underscoring the complex interplay between acetylation and phosphorylation in the modulation of the PTM landscape of tau (Cook et al., 2014). Notably, sirtuin 1 levels are reduced, and HDAC6 levels are elevated in AD brains, reflecting their distinct contributions to the progression of the disease (Carlomagno et al., 2017).

##### Methylation

Tau methylation, which occurs on both lysine and arginine residues, has been observed in a significant portion of NFTs in the postmortem brains of AD patients (Thomas et al., 2012). Specifically, seven lysine residues (K44, K163, K174, K180, K254, K267, and K290) within the projection and MBRD domains of paired helical filament (PHF) tau derived from the AD brain were identified as methylation sites (Thomas et al., 2012). Methylation can occur on lysine residues as either a mono- or dimethyl modification (Funk et al., 2014). Additionally, monomethylation of arginine residues R126, R155, and R349 was observed in tau from both wild-type and human APP transgenic mice (Morris et al., 2015). This methylation process was found to reduce the aggregation of recombinant tau proteins while not affecting their ability to support microtubule assembly (Funk et al., 2014). The protein lysine methyltransferase SETD7 selectively monomethylates tau protein at K132. Furthermore, K132-monomethylated tau proteins are primarily localized within the cell soma and nuclear compartments and are absent from neurites (Bichmann et al., 2021).

##### Glycosylation

Glycosylation has been identified at 32 lysine residues across the entire length of the tau protein (Nacharaju et al., 1997). Research has shown that both *N*- and *O*-glycosylation occur in tau (Arnold et al., 1996; Wang et al., 1996). Notably, *N*-glycosylation has been specifically observed in PHF tau extracted from postmortem AD brains, distinguishing it from the tau found in healthy individuals (Wang et al., 1996). *N*-glycosylation is associated with an increased tendency for tau to become hyperphosphorylated (Liu et al., 2002), but interestingly, it also leads to a reduction in tau aggregation (Losev et al., 2019). On the other hand, *O*-GlcNAcylation, a particular type of *O*-glycosylation, seems to offer a protective effect against tau-related pathologies. There is a notable decrease in *O*-GlcNAcylated tau in the brains of AD patients. Additionally, experiments have shown that blocking *O*-GlcNAcylation leads to an increase in tau phosphorylation in the rat brain, suggesting a critical role in regulating pathological tau modifications (Liu et al., 2009).

##### Truncation

Proteolytic cleavage of tau by various enzymes, including caspases, asparagine endopeptidase (AEP), calpains, and ADAM10, results in the generation of truncated tau species (Guo et al., 2020). Caspases 2 and 3 cleave tau at D314 and D421, generating truncated Δtau-314 and Δtau-421, respectively. These truncated proteins have been found at elevated levels in the postmortem brains of AD patients (Gamblin et al., 2003; Zhao et al., 2016b). Cleavage at D314 is critical for the mislocalization of both full-length tau and Δtau-314 to dendritic spines, a necessary step for tau-P301L to trigger neurodegeneration in mice expressing EGFP-tau-P301L (D314E). However, Δtau-314 alone does not impair synaptic function in mice expressing EGFP-Δtau-314. Interestingly, reducing caspase-2 levels was shown to restore memory function in rTg4510 mice (Zhao et al., 2016b). Moreover, Aβ promotes tau cleavage at D421 via caspase-3 and -7, representing an early pathological event in AD, with Δtau-421 facilitating the assembly of tau filaments *in vitro*, and these filaments in AD brains show immunoreactivity to NFTs (Gamblin et al., 2003; Rissman et al., 2004). Additionally, caspase-6 cleaves tau at residues D13 and D402, generating Δtau-13 and Δtau-402, respectively (Horowitz et al., 2004). The presence of active caspase-6 and truncated tau forms (Δtau-13 and -402) is significant in AD and, to a lesser extent, in PiD but not in argyrophilic grain disease (AGD), CBD, or PSP. These findings suggest that caspase-6 is a potential therapeutic target for AD and possibly PiD (Theofilas et al., 2022).

AEP, a lysosomal cysteine proteinase, becomes activated with aging and in the brains of individuals with AD and P301S mice (Zhang et al., 2014). AEP cleaves tau at residues N167, N255, and N368 (Behrendt et al., 2019; Zhang et al., 2014). Depletion of AEP reduced tau hyperphosphorylation and ameliorated synaptic and cognitive deficits in PS19 mice. The ability of truncated tau proteins (amino acids 1–255 and 1–368) to stabilize microtubules is disrupted, facilitating tau hyperphosphorylation and aggregation. Inhibiting AEP cleavage prevents the neurodegeneration induced by tau P301S in PS19 mice expressing mutant tau-P301S (N255A, N368A) (Zhang et al., 2014).

#### Tau seeding and spreading

NFTs consist of PHFs and straight tau filaments (SFs), as identified in early studies (Kidd, 1963; Terry, 1963; Yagishita et al., 1981). Recent advancements in cryo-EM have revealed that the core of tau filaments (amino acids 306–378 of tau protein) is formed from two identical protofilaments. These protofilaments exhibit a unique cross-β/β-helix structure, acting as nucleation sites for further tau aggregation. Notably, PHFs and SFs differ in how their protofilaments are arranged (Fitzpatrick et al., 2017). Various tauopathies, including AD, chronic traumatic encephalopathy (CTE), CBD, PiD, and PSP, are characterized by distinct tau fold patterns, highlighting the diversity of tau pathology (Falcon et al., 2018a, 2018b, 2019; Fitzpatrick et al., 2017; Shi et al., 2021b; Zhang et al., 2020). Interestingly, NFTs in primary age-related tauopathies are similar to those in AD (Shi et al., 2021a).

Tau aggregation is influenced by a complex interplay of PTMs, genetic mutations, and specific polymerization inducers, leading to the formation of toxic NFTs within neurons (Congdon and Sigurdsson, 2018). This contributes significantly to neurodegeneration. It has been suggested that smaller, soluble tau oligomers are more detrimental to cellular health than larger, mature filaments are (Cardenas-Aguayo Mdel et al., 2014; Shafiei et al., 2017).

In AD, NFT pathology begins in the transentorhinal and entorhinal cortex, advancing in a predictable pattern through the hippocampus and into the neocortex. The propagation of tau pathology, as determined by Braak staging in postmortem studies, correlates with disease severity (Braak and Braak, 1995; Braak et al., 2006). Modern imaging technologies, such as positron emission tomography (PET) and MRI, have confirmed the anticipated spread of tau pathology and its association with brain functional reorganization, supporting the hypothesis of transneuronal tau propagation (Cope et al., 2018). In primary age-related tauopathy, NFTs predominantly affect the medial temporal lobe and other specific brain regions (Crary et al., 2014).

The transmission of tau from cell to cell involves the self-propagation of proteopathic tau seeds, which trigger the accumulation and templated fibrillization of endogenous tau, spreading abnormal tau across the brain in a manner reminiscent of prions (Gibbons et al., 2019; Walker and Jucker, 2015). According to experimental models, intracerebral injection of brain extracts containing tau aggregates into tau transgenic mice induces NFT formation and pathological spread (Ahmed et al., 2014; Clavaguera et al., 2009), a phenomenon also observed with brain extracts from human tauopathies (Boluda et al., 2015; Clavaguera et al., 2013). Intriguingly, peripheral injection of tau aggregates can also trigger tauopathy in transgenic mice (Clavaguera et al., 2014).

Extracellular tau, both exosome-associated and freely soluble, plays a role in the dissemination of tau pathology (Asai et al., 2015; Fiandaca et al., 2015; Jia et al., 2019; Kanmert et al., 2015; Saman et al., 2012; Wang et al., 2017b). Neurons and microglia release tau-containing exosomes, facilitating the spread of tau pathology (Asai et al., 2015; Mothes et al., 2023; Wang et al., 2017b). Pathological tau is taken up by interconnected neurons or adjacent glial cells via endocytosis, micropinocytosis, or direct membrane fusion (Asai et al., 2015; Calafate et al., 2016; Christianson and Belting, 2014; Frost et al., 2009; Mulcahy et al., 2014; Wu et al., 2013). Low-molecular-weight (LMW) tau aggregates and short fibrils are particularly prone to endocytosis and subsequent neuron-to-neuron transmission (Frost et al., 2009; Wu et al., 2013). Heparan sulfate proteoglycans (HSPGs) also play a crucial role in the binding, internalization, and propagation of tau (Holmes et al., 2013).

A key step in the spread of tau fibrils involves endolysosomal damage (Gibbons et al., 2019). Disrupting lysosomal function with chloroquine reduces the degradation of synthetic tau preformed fibrils (PFFs) and increases the aggregation of endogenous tau in neurons expressing Tau P301L-GFP (Gibbons et al., 2017). Furthermore, the deletion of PI4K2A, a kinase in the phosphoinositide-initiated membrane tethering and lipid transport (PITT) pathway essential for lysosomal repair, exacerbates tau fibril spreading in cell-based assays (Tan and Finkel, 2022).

Increasing evidence indicates that the deposition of Aβ plaques is essential for the propagation of tau pathology in AD. Aβ plaques facilitate the rapid amplification of pathological tau derived from human AD brain extracts and promote the formation of large tau aggregates. This process subsequently triggers the formation and dissemination of NFTs and neuropil threads (NTs) in *App*^*NL-G-F*^-knock-in and 5× FAD transgenic mouse models (He et al., 2018).

### Neuronal loss

Extensive loss of neurons, especially in the regions critical for memory and higher cognitive functions, is a hallmark of AD (DeTure and Dickson, 2019; Serrano-Pozo et al., 2011). In the healthy adult brain, mature neurons utilize intricate mechanisms to suppress the activation of cell death signaling (Kole et al., 2013). However, in the context of AD, these protective mechanisms appear to be compromised, leading to the aberrant activation of various forms of regulated cell death (RCD), including necroptosis, pyroptosis, apoptosis, ferroptosis, and autophagy-dependent cell death (Goel et al., 2022; Thal et al., 2024). Neurotoxic Aβ species trigger synaptic dysfunction and neuronal death through ectopic cell cycle reentry, a process dependent on tau (Bloom, 2014). Cell cycle reentry is an early and critical event contributing to neuronal loss in AD (Fricker et al., 2018; Yang et al., 2003).

The complex interplay among various RCD cascades is believed to be the primary driver of neuronal death in AD (Goel et al., 2022; Thal et al., 2024). Necroptosis has been detected in the brains of postmortem AD patients and is positively associated with tau pathology (Caccamo et al., 2017). The neuron-specific long non-coding RNA *MEG3*, which is upregulated in AD patients, contributes to neuronal necroptosis. In a xenograft AD model using human neurons transplanted into *Rag2*^−/−^/*App*^*NL-G-F*^ transgenic mice, the downregulation of *MEG3* or the inhibition of necroptosis rescued the neurons from death (Balusu et al., 2023). Both NLRP1 and NLRP3 inflammasomes, which drive pyroptosis, are activated in AD patients (Saresella et al., 2016). Knockdown of *Nlrp1* or *Casp1* has been shown to reduce neuronal pyroptosis and enhance cognitive function in the APPswe/PS1dE9 mouse model (Tan et al., 2014). Elevated iron levels and lipid peroxidation, characteristic of ferroptosis, have been observed in AD pathology (Yan et al., 2021). Blocking ferroptosis has been shown to mitigate neuronal loss and cognitive impairments associated with Aβ and tau toxicity (Bao et al., 2021; Zhang et al., 2018). Furthermore, the accumulation of Aβ and tau is believed to induce apoptosis, contributing to neuronal loss and AD progression (Kumari et al., 2023; Wu et al., 2024). However, several studies have reported that histological evidence of neurons displaying the characteristic morphology of apoptosis is surprisingly rare in the postmortem brains of AD patients (Lucassen et al., 1997; Perry et al., 1998; Stadelmann et al., 1998). This discrepancy might be explained by survivor bias, where neurons that have undergone apoptosis and been cleared are not present in postmortem analyses. Moreover, impaired autophagy in AD brain has been associated with neuronal senescence and intraneuronal Aβ accumulation (Lee et al., 2022; Nixon, 2007; Suelves et al., 2023), which contrasts with the physiological role of autophagy in preventing the accumulation of misfolded proteins. Additionally, death induced by survival gene elimination (DISE) is a cell death mechanism activated by short RNAs (sRNAs) with specific 6-nucleotide seed sequences. In AD mouse models and iPSC-derived neurons from AD patients, RNA-induced silencing complex (RISC)-bound sRNAs shift to more toxic 6-mer seed sequences. Inhibition of RISC activity or genetic deletion of *Ago2* attenuates Aβ_42_-induced neuronal cell death and DNA damage (Paudel et al., 2024).

These studies suggest that targeting cell death mechanisms may be a potential therapeutic approach to protect neurons and mitigate neurodegeneration in AD. However, the precise triggers and mechanisms underlying RCD remain elusive, necessitating further research to identify specific pathways and their therapeutic potential. The development of suitable imaging techniques to detect neuronal death in the ante-mortem brain could provide valuable insights into this critical aspect of AD pathogenesis.

### Demyelination

Myelin, a glial membrane tightly wrapped around axons in a spiral fashion, plays a crucial role in enhancing conduction speed. Its less compact regions enable oligodendrocytes to support the metabolic needs of neurons (Saab et al., 2016). Studies utilizing macroscopic brain imaging have revealed early signs of cortical myelin damage in patients with AD during its preclinical phase, suggesting that these changes are potential early indicators of brain pathology (Araque Caballero et al., 2018; Dean et al., 2017; Ringman et al., 2007). Furthermore, abnormalities specific to certain brain regions involved in myelination were detected before the emergence of amyloid and tau pathology in a triple-transgenic AD mouse model (Desai et al., 2009). A reduction in oligodendrocyte numbers has also been observed in the brains of AD patients after death (Behrendt et al., 2013).

Single-cell transcriptomics of AD mouse models revealed distinct changes in oligodendrocytes, especially near amyloid plaques (Chen et al., 2020; Kenigsbuch et al., 2022). Moreover, RNA sequencing of postmortem AD samples revealed that the expression of myelin-related genes was significantly altered, indicating that myelination and demyelination processes are key factors in AD progression (Mathys et al., 2019, 2023; Zhou et al., 2020). Oligodendrocytes in individuals with AD are notably more susceptible to DNA damage (Mathys et al., 2023). A specific oligodendrocyte population, referred to as disease-associated oligodendrocytes (DAOs), was identified in both AD mouse models and AD patients and plays a pivotal role in disease pathology (Kenigsbuch et al., 2022; Pandey et al., 2022). Targeting DAOs has been shown to improve axonal myelination, reduce Aβ-related pathologies, and slow cognitive decline in an AD mouse model (Park et al., 2023).

Interestingly, individuals with multiple sclerosis (MS) have a greater likelihood and increased risk of being diagnosed with AD or dementia (Mahmoudi et al., 2022). Experiments have demonstrated that myelin dysfunction and demyelinating injuries can accelerate Aβ plaque accumulation in models of experimental autoimmune encephalomyelitis (EAE) and in cuprizone-treated AD mice (Depp et al., 2023). These findings highlight the importance of focusing on oligodendrocyte health and myelin integrity as potential strategies to slow AD progression.

### Gliosis and neuroinflammation

**Figure 3.**
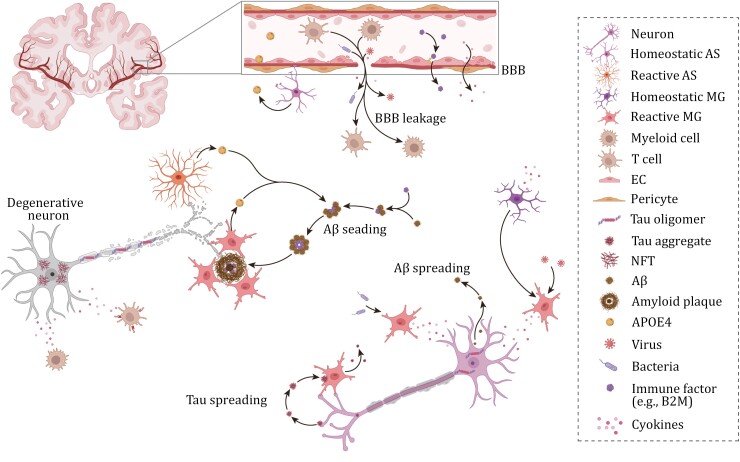
Impact of systemic inflammation on AD pathogenesis. Beyond Aβ and tau, systemic inflammation, triggered by chronic conditions (such as obesity, diabetes, cardiovascular and cerebrovascular diseases) and microbial infections (including those caused by bacteria and viruses), compromises the BBB. This disrupted BBB integrity allows peripheral immune cells (such as T cells and myeloid cells), along with proinflammatory cytokines and microbes, to penetrate the brain parenchyma. This invasion initiates a chain of inflammatory responses that leads to the activation of reactive microglia and astrocytes, thereby accelerating the formation of amyloid plaques and tau pathology, which results in neuronal degeneration. Consequently, this cascade significantly contributes to AD progression. Abbreviations: Aβ, β-amyloid; AS, astrocyte; B2M, β2-microglobulin; BBB, blood‒brain barrier; EC, endothelial cell; MG, microglia; NFT, neurofibrillary tangle.

Accumulating evidence indicates that reactive astrogliosis and microgliosis are significant pathological features of AD and play critical roles in its pathogenesis (Fig. 3). *APOEε4*, the most significant genetic risk factor for sporadic AD, can increase the risk of developing the disease by 3–15 times. This gene is predominantly expressed by astrocytes and microglia within the CNS (Jansen et al., 2019). Moreover, a variety of single nucleotide polymorphisms (SNPs) and rare coding variants in genes related to the immune system, which are thought to influence microglial function, have been recognized as risk factors for AD. These genes include *TREM2*, *BIN1*, *CLU*, *CR1*, *PICALM*, *CD33*, and the *MS4A* gene cluster, which were identified through WGS and GWAS (Karch and Goate, 2015).

#### Microglia

Microglia, the brain’s resident immune cells, are thought to originate from primitive macrophages that arise from erythromyeloid precursors in the yolk sac, contributing to the brain’s innate immune system throughout life (Ginhoux et al., 2010; Gomez Perdiguero et al., 2015; Kierdorf et al., 2013). A specific subset of microglia, termed repair-associated microglia (RAMs), has been identified for its role in repairing damaged brain vasculature and facilitating functional recovery post-injury (Choi et al., 2023; Mastorakos et al., 2021).

In the AD brain, reactive microglia cluster around amyloid plaques, suggesting a crucial relationship between these immune cells and one of the hallmark pathologies of AD (McGeer et al., 1987; Tooyama et al., 1990). This association extends to pathological tau, with studies showing a link between tau accumulation and microglial activation (Dani et al., 2018; Hayes et al., 2002). Disease-associated microglia (DAMs) have been discovered in models such as 5× FAD mice, where they localize near amyloid plaques and potentially limit neurodegeneration. The transition to DAM involves a TREM2-independent phase characterized by downregulation of homeostatic genes and upregulation of genes involved in phagocytosis and lipid metabolism in a TREM2-dependent manner (Keren-Shaul et al., 2017). Lipid droplet-accumulating microglia (LDAM) are abundant in AD patients carrying *APOEε4/ε4*. Furthermore, fibrillar Aβ has been shown to induce lipid droplet accumulation in an APOE-dependent manner (Haney et al., 2024). Additionally, terminally inflammatory microglia (TIMs) and dystrophic (senescent) microglia, which are associated with impaired Aβ clearance and tau pathology, respectively, have been identified, highlighting the diverse roles of microglia in AD (Millet et al., 2024; Streit et al., 2009).

Research led by Kellis and Tsai into the transcriptomic and epigenomic landscape of AD has shown that noncoding AD risk loci are uniquely accessible in microglia, with an increase in lipid processing and inflammatory microglia correlating with disease progression and severity (Xiong et al., 2023). The spatial and disease stage-dependent morphological heterogeneity of microglia underscores the complexity of their roles in AD, with changes in shape and function reflecting their proximity to plaques and disease progression (Plescher et al., 2018). These findings highlight the need for careful assessment of the spatial and temporal variations in microglial populations.

Microglia express various Aβ receptors, including TREM2, LRP1, TLRs, CR3, CD14, CD47, CD36, α6β1 integrin, and RAGE (Doens and Fernandez, 2014). These receptors facilitate microglial interactions with Aβ, influencing processes such as phagocytosis, inflammation, and clearance of Aβ. For instance, CR3 plays a role in microglia-mediated phagocytosis and clearance of Aβ in conjunction with complement C3 (Fu et al., 2012). Exposure to soluble Aβ oligomers triggers reactive microglia to engulf synapses in a CR3-dependent manner (Hong et al., 2016). Deactivating CR3 has been shown to reduce tau pathology and microglial phagocytosis of synapses in PS19 mice (Litvinchuk et al., 2018), while a separate study revealed that CR3 ablation decreased Aβ deposition in T41 APP-transgenic mice (Czirr et al., 2017). Moreover, TLR2 binds to oligomeric Aβ_42_ aggregates, increasing Aβ_42_-induced inflammation but decreasing Aβ phagocytosis by cultured microglia (Liu et al., 2012). Notably, the microglial lysosomal system plays a crucial role in the propagation of both Aβ and tau pathology (Bao et al., 2016; Majumdar et al., 2011; Van Acker et al., 2021; Wang et al., 2024).

The inflammatory response mediated by microglia is pivotal in the development of AD pathology. Notably, the activation of the NLRP3 inflammasome in microglia, triggered by Aβ, leads to caspase-1 activation and IL-1β maturation, playing a significant role in the pathogenesis of AD (Heneka et al., 2013). Moreover, the presence of the NLRP3 inflammasome is essential for the development of Aβ-induced tau pathology, with its absence reducing tau pathology and ameliorating cognitive deficits in Tau22 transgenic mice (Ising et al., 2019). The inhibition of microglial NF-κB signaling has also been demonstrated to alleviate Aβ neurotoxicity and limit the spread of tau (Chen et al., 2005; Wang et al., 2022).

Reactive microglia are crucial for the transmission of pathological tau, facilitating its spread from neuron to neuron via phagocytosis and subsequent exocytosis within exosomes. The use of the CSF1R inhibitor PLX3397 to deplete microglia markedly decreased the progression of tau pathology in mouse models, including those injected with adeno-associated virus (AAV)-GFP-tau and PS19-tau transgenic mice (Asai et al., 2015). Microglia also play a role in modulating Aβ pathology; continuous microglial depletion through the CSF1R inhibitor PLX5622 led to a reduction in amyloid plaque deposition in a 5× FAD mouse model (Spangenberg et al., 2019). However, a contrasting study using PLX3397 for microglial ablation showed similar amyloid plaque burdens between the control and treated groups, with an increase observed in 5× FAD mice following microglial repopulation (Gratuze et al., 2021). Additionally, depletion or repopulation of microglia significantly affected tau seeding and spreading in neurons adjacent to amyloid plaques in 5× FAD mice injected with tau aggregates from human AD brain extracts (Gratuze et al., 2021).

Dysregulated innate and adaptive immune responses are implicated in AD pathogenesis (Hammond et al., 2019; Heneka et al., 2014). Specifically, an increase in cytotoxic T cells has been observed in brain regions affected by tauopathy in both tau transgenic mice and individuals with AD. Depletion of microglia via PLX3397 was shown to prevent T-cell infiltration in human *APOEε4*-knock-in PS19 (TE4) mice (Chen et al., 2023), underscoring the critical interaction between microglia and the adaptive immune system in AD progression. In contrast to the long-standing belief that only adaptive immune cells possess immunological memory, emerging evidence indicates that myeloid cells, including microglia, also exhibit memory-like responses. For instance, a single peripheral administration of low-dose lipopolysaccharide (LPS) exacerbates brain inflammation and amyloid deposition in the APP23 transgenic mouse model. However, repeated LPS injections induce immune tolerance, which in turn mitigates amyloid pathology (Wendeln et al., 2018). These findings suggest that targeting the memory-like properties of microglia may offer potential therapeutic avenues for AD treatment.

#### Astrocyte

Astrocytes, a major glial cell type in the CNS derived from neural progenitor cells, play a vital role in maintaining extracellular fluid and neurotransmitter homeostasis, inducing synapse formation, and providing metabolic and neurotrophic support for synapses (Brandebura et al., 2023; Leng and Edison, 2021). Increased reactive astrocytes around amyloid plaques are noted in the postmortem brains of AD patients (Beach and McGeer, 1988).

Astrocyte heterogeneity in AD patients indicates differential expression across cortical layers (Sadick et al., 2022), and a distinct subpopulation of disease-associated astrocytes (DAAs) identified in 5× FAD model mice and aging human brains suggests accelerated astrocyte aging in AD (Habib et al., 2020).

Astrocytes also play a key role in the glymphatic system, facilitating interstitial fluid clearance via aquaporin 4 (AQP4). Disruption of this pathway exacerbates Aβ accumulation and cognitive deficits, highlighting the importance of astrocytic AQP4 in AD pathogenesis (Iliff et al., 2012; Xu et al., 2015). Astrocyte reactivity is increasingly recognized as a diverse response within the brain. Notably, certain reactive astrocytes serve a neuroprotective function, slowing the progression of AD. The elimination of these astrocytes in *Gfap* and *Vim* double-knockout APP/PS1 mice led to an increase in amyloid plaque accumulation and associated neuronal damage (Kraft et al., 2013). Furthermore, the activation of reactive astrocytes through astrocyte-specific *Nrf2* expression has been shown to decrease amyloid deposits and phosphorylated tau levels, ameliorating cognitive impairments in both APP/PS1 and Thy1-hTau P301S mouse models (Jiwaji et al., 2022). This finding underscores the critical impact of reactive astrocytes in limiting the spread of amyloid and tau pathology. Conversely, when activated by neuroinflammatory cytokines such as IL1α, TNFα, and C1q, reactive astrocytes can become detrimental, leading to neuron and oligodendrocyte death rather than promoting neuronal health and connectivity (Liddelow et al., 2017).

In studies using human iPSC-derived astrocyte cultures, these cells were observed to engulf neuronal debris, promoting cell-to-cell propagation of tau pathology. This process suggests that once astrocytes internalize tau, they might become harmful to adjacent neurons (Mothes et al., 2023). The transcription factor TFEB, a master regulator of lysosome biogenesis, was elevated in the brains of deceased AD and FTD patients and in rTg4510 tau transgenic mice. Increasing TFEB expression boosts lysosomal function and increases the uptake of tau fibrils by primary astrocytes. Additionally, astrocytic TFEB expression can hinder the spread of tau pathology in PS19-tau transgenic mice (Martini-Stoica et al., 2018). In contrast, disrupting the interaction between TFEB and the v-ATPase impairs lysosomal function and exacerbates tau pathology in PS19 mice (Wang et al., 2024), underscoring the pivotal role of astrocytes in modulating tau pathology.

However, the interaction with pathological tau oligomers triggers the release of high mobility group box 1 (HMGB1), a nuclear protein involved in DNA processes, leading to astrocyte senescence. Blocking HMGB1 release not only decreased the number of senescent astrocytes but also reduced tau tangle formation in tauopathy mouse models (Gaikwad et al., 2021). This finding suggested that a decrease in astrocyte surveillance may be linked to accelerated senescence during AD progression.

## Risk factors

### Genetic risk factors

#### APOE

The *APOE* gene, located on chromosome 19 in humans, is characterized by three allelic forms: *APOEε2*, *APOEε3*, and *APOEε4*. These variants are distinguished by differences at amino acid positions 112 and 158. Specifically, APOE2 carries cysteine at both positions 112 and 158, APOE3 has cysteine at position 112 and arginine at 158, and APOE4 features arginine at both positions (Yamazaki et al., 2019). These subtle variations in single amino acids significantly influence APOE’s structure and function, affecting its ability to bind to lipids and receptors (Yamazaki et al., 2019). For instance, APOE3 and APOE4 exhibit a high affinity for the low-density lipoprotein receptor (LDLR), in contrast to APOE2, which shows a markedly reduced binding affinity, which is 50 to 100 times lower (Weisgraber et al., 1982).

APOE plays a crucial role in the transport of cholesterol and other lipids to neurons through its interaction with cell surface receptors such as LDLR and LDLR-related protein 1 (LRP1) (Bu, 2009). APOE4 is associated with increased AD risk through mechanisms such as enhanced Aβ aggregation, intraneuronal Aβ accumulation, early Aβ seeding, amyloid plaque formation, and CAA pathology (Christensen et al., 2010; Koffie et al., 2012; Kok et al., 2009; Liu et al., 2017; Polvikoski et al., 1995; Rannikmae et al., 2014; Schmechel et al., 1993; Shinohara et al., 2016; Tiraboschi et al., 2004). In contrast, APOE2 has been shown to protect against the progression of Aβ pathology over time (Chiang et al., 2010; Grothe et al., 2017b; Lim et al., 2017; Millet et al., 2024; Morris et al., 2010).

In studies involving APOE-targeted replacement of APP-V717F transgenic mice, APOE4 was shown to be less effective at clearing Aβ from the interstitial fluid (ISF) than APOE2 and APOE3 (Castellano et al., 2011). APOE4 alters the preferred pathway for rapid Aβ clearance from being mediated by LRP1 to involving the very low-density lipoprotein receptor (VLDLR), leading to a slower rate of Aβ internalization and efflux at the BBB (Deane et al., 2008). Moreover, APOE, when interacting with LRP1, inhibits Aβ uptake in astrocytes rather than directly binding to Aβ (Verghese et al., 2013). Additionally, APOE4 has been found to be less effective in facilitating the microglial uptake and degradation of Aβ than APOE2 and APOE3 (Jiang et al., 2008).

APOE4 is known to worsen tau pathology, neuroinflammation, and brain atrophy in APOE4-targeted replacement PS19 mice compared to APOE2 and APOE3 variants (Shi et al., 2017). Furthermore, when APOE4 is expressed by microglia, it disrupts lipid metabolism, impairs microglial function, and reduces the ability of microglia to respond to AD pathology. On the other hand, the microglial expression of APOE3 is associated with increased proximity of microglia to amyloid plaques, a reduction in amyloid pathology, and an improvement in cognitive function. Removing APOE4 from microglia restores the response to chronic neurodegeneration and lessens AD pathology in both PS19-tau transgenic mice and APP/PS1 mice, highlighting the critical role of microglial APOE in AD (Liu et al., 2023a; Yin et al., 2023).

APOE is highly expressed in both the brain and peripheral tissues. In the periphery, it is mainly produced by hepatocytes, with a plasma concentration of approximately 40–70 μg/mL (Elshourbagy et al., 1985). A higher ratio of plasma APOE4 to APOE3 has been linked to regional brain volume loss, decreased cerebral glucose metabolism, and impaired cognitive performance (Nielsen et al., 2017). The expression of human *APOEε4* in the liver showed a toxic gain-of-function effect, impairing synaptic plasticity and cognitive functions by affecting cerebrovascular health, even in the absence of brain-expressed APOE in *Apoe*-null mice. Moreover, plasma from young mice with *APOEε3*, when transfused into older mice, improved cognition and reduced vessel-associated gliosis. Conversely, plasma containing APOE4 negated the beneficial effects of young plasma, underscoring the significant impact of peripheral APOE on AD pathogenesis (Liu et al., 2022).

Notably, two missense variants of *APOE*—*APOEε3*-V236E and *APOEε4*-R251G—are associated with a 2- to 3-fold reduced risk of AD (Le Guen et al., 2022). Additionally, research involving the largest known family with autosomal dominant AD, carrying the Colombian *PSEN1* E280A mutation, has revealed potentially protective effects against autosomal dominant AD, including homozygosity for the *APOEε3* Christchurch (R136S) mutation (Arboleda-Velasquez et al., 2019) and heterozygosity for the *RELN* COLBOS (H3447R) mutation (Lopera et al., 2023). Individuals with these mutations exhibited limited tau pathology and a delayed onset of autosomal dominant AD symptoms despite a high burden of Aβ plaques. In particular, the *APOEε3*-R136S mutation enhanced the microglial response to amyloid plaques and reduced Aβ-induced tau seeding and spreading in human *APOEε3*-R136S knock-in APP/PS1 mice injected with tau fibrils from human AD brain extracts (Chen et al., 2024). Similarly, the *APOE*-R136S mutation alleviated *APOEε4*-associated tau pathology, neurodegeneration, and neuroinflammation in human *APOEε4*-R136S-knock-in PS19 mice (Nelson et al., 2023). These findings highlight the protective potential of the *APOEε3*-R136S and *RELN*-H3447R mutations against AD pathologies, offering a promising avenue for therapeutic development aimed at preventing cognitive decline and dementia in AD patients.

#### TREM2

The triggering receptor expressed on myeloid cells 2 (TREM2) gene is located on chromosome 6 in humans. TREM2, exclusively expressed in microglia within the CNS, is a single-transmembrane immune receptor of the immunoglobulin superfamily (Deczkowska et al., 2020). The heterozygous TREM2-R47H variant significantly increases the risk of AD by 3–4 times (Guerreiro et al., 2013; Jonsson et al., 2013).

The transmembrane helix within TREM2 interacts with the adaptor protein DAP12, which is crucial for TREM2 membrane stabilization and the initiation of downstream signaling pathways (Peng et al., 2010; Zhong et al., 2015). Variants linked to AD affect the expression of TREM2, its trafficking to the cell surface, its shedding, its ligand binding, and its downstream signaling (Jiang et al., 2016; Jin et al., 2014; Olive et al., 2020; Parhizkar et al., 2019; Roussos et al., 2015; Song et al., 2017).

The extracellular immunoglobulin-like domain of TREM2 recognizes various pathological molecules in AD, including phospholipids (Wang et al., 2015), lapidated particles (Song et al., 2017), APOE (Atagi et al., 2015; Bailey et al., 2015; Yeh et al., 2016), Aβ (Zhao et al., 2018; Zhong et al., 2018), TDP-43 (Xie et al., 2022), galectin-3 (Boza-Serrano et al., 2019), and C1q (Zhong et al., 2023). For example, the binding of microglial TREM2 to complement C1q restricts complement-mediated synaptic engulfment, thereby reducing synaptic loss in PS19 tau transgenic mice (Zhong et al., 2023).

*Trem2* deficiency results in the inability of microglia to cluster around Aβ plaques, leading to increased Aβ accumulation in 5× FAD mice (Wang et al., 2015). This deficiency disrupts microglial barrier function, affecting amyloid compaction and leading to the formation of longer, branched amyloid fibrils (Yuan et al., 2016). Furthermore, 5× FAD mice with the TREM2 R47H variant exhibit loss-of-function defects, including impaired microglial clustering and altered plaque morphology (Song et al., 2018). AD-associated TREM2 variants also show a reduced affinity for Aβ, which is crucial for initiating TREM2-mediated signaling that promotes microglial migration and clustering (Zhao et al., 2018; Zhong et al., 2018). In contrast, a distinct subpopulation of senescent microglia with elevated TREM2 expression was identified in the 5× FAD mouse model. Selective elimination of these senescent microglia subtypes, but not the DAM population, led to improved cognitive performance (Rachmian et al., 2024). This finding highlights the complex role of TREM2 in regulating distinct microglial phenotypes, which should be carefully considered when evaluating TREM2 as a therapeutic target for AD.

*Trem2* deficiency exacerbates tau pathology in a humanized tau mouse model (Bemiller et al., 2017). While *Trem2* haploinsufficiency intensifies tau pathology and brain atrophy in PS19 mice, complete *Trem2* deficiency surprisingly protects against tau-mediated microglial activation and brain atrophy (Sayed et al., 2018), although some studies have reported no effect of *Trem2* deficiency on tau pathology in PS19 mice (Leyns et al., 2017).

TREM2 undergoes proteolytic cleavage by ADAM10/17 at residue H157, releasing its ectodomain as a soluble form (sTREM2) into the extracellular space (Feuerbach et al., 2017; Schlepckow et al., 2017; Thornton et al., 2017). The TREM2 H157Y variant is associated with increased AD risk (Jiang et al., 2016). Conversely, elevated levels of sTREM2 in the CSF correlate with reduced AD risk (Deming et al., 2019), suggesting a protective role for sTREM2. sTREM2 appears to mitigate AD pathology by supporting microglial survival, promoting the release of inflammatory cytokines, enhancing microglial clustering around amyloid plaques, and facilitating microglial uptake and degradation of Aβ (Zhong et al., 2017, 2019).

#### Trisomy 21

DS is the most common form of intellectual disability, resulting from a complete or partial triplication of chromosome 21. Globally, DS affects approximately one in every 1000 newborns, with an estimated 5.4 million individuals living with DS worldwide (GBD 2015 Disease and Injury Incidence and Prevalence Collaborators, 2016). By the age of 40, all individuals with DS will have developed neuropathological features characteristic of AD, making trisomy 21 the most significant risk factor for early-onset AD (Leverenz and Raskind, 1998; Lott and Head, 2019; Wisniewski et al., 1985). Research indicates that the levels of Aβ and tau proteins, both of which are implicated in AD, increase with age in the brains of individuals with DS (Condello et al., 2022; Janelidze et al., 2022).

Furthermore, trisomy 21 exacerbates neuroinflammation and the accumulation of amyloid plaques in DS-AD combined mouse models. These studies pinpointed the chromosome 21-encoded deubiquitinase USP25 as a crucial factor in the development of AD. The genetic deletion or pharmacological inhibition of USP25 was shown to restore microglial homeostasis, curb the release of microglia-driven cytokines and synaptic phagocytosis, diminish Aβ plaque accumulation, and improve synaptic and cognitive functions in AD mouse models (Zheng et al., 2021, 2022). These findings illuminate how trisomy 21 influences the pathogenesis of DS and AD, suggesting that USP25 is a viable therapeutic target for both conditions.

#### Other risk genes

The bridging integrator 1 (BIN1) gene, located on chromosome 2 in humans, has been identified as a critical risk gene for sporadic AD, ranking second in significance only to APOE according to the AlzGene database (Bertram et al., 2007). Research indicates that higher levels of BIN1 in the brains of individuals with AD are linked to a later onset of the disease (Karch et al., 2012). Notably, the SNPs rs59335482 and rs744373 in *BIN1* are associated with an increase in tau pathology but not with Aβ pathology (Chapuis et al., 2013; Franzmeier et al., 2019). Overexpression of BIN1 has been shown to mitigate tau aggregation and reverse deficits in long-term memory in tau transgenic mice (Sartori et al., 2019). However, a deficiency in BIN1 facilitates the spread of tau pathology (Calafate et al., 2016). In contrast, a study involving Drosophila demonstrated that reducing the expression of *Amph*, the equivalent of *BIN1* in flies, decreased tau-related neurotoxicity (Chapuis et al., 2013). Moreover, ablation of *Bin1* specifically in microglia reduced tau spread and hyperphosphorylation in male PS19 transgenic mice (Crotti et al., 2019).

Clusterin (CLU), also known as apolipoprotein J (APOJ), is predominantly an extracellular chaperone (Jones and Jomary, 2002) and is located on chromosome 8 in humans. Elevated plasma levels of clusterin have been significantly linked to both the onset and severity of AD (Schrijvers et al., 2011). The ability of clusterin to bind and sequester Aβ oligomers plays a crucial role in hindering their growth or dissociation (Narayan et al., 2011). The overexpression of clusterin in astrocytes has been shown to reduce Aβ pathology and ameliorate synaptic deficits in 5× FAD mice (Chen et al., 2021). Conversely, CLU depletion was found to decrease fibrillar Aβ deposits and dystrophic neurites in APP-V717F transgenic mice (DeMattos et al., 2002). Another study noted that while CLU deficiency led to fewer Aβ plaques in the brain parenchyma, it surprisingly resulted in an increased presence of CAA within the cerebrovasculature in APP/PS1 transgenic mice (Wojtas et al., 2017).

CD33, or sialic acid-binding immunoglobulin-like lectin 3 (Siglec-3), is mainly expressed on microglia, monocytes, and macrophages and plays roles in cell adhesion, endocytosis, and the immune response (Crocker et al., 2007). Increased CD33 expression in microglia in the AD brain is positively correlated with amyloid plaque accumulation. Deleting *CD33* has been shown to enhance microglial Aβ uptake and reduce Aβ plaque formation in APP/PS1 transgenic mice (Griciuc et al., 2013). Moreover, the absence of CD33 boosts the phagocytosis of Aβ oligomers by microglia (Wissfeld et al., 2021). Additionally, CD33 deficiency has been shown to decrease Aβ pathology and improve cognitive function in 5× FAD;*CD33*^−/−^ mice. However, these benefits are negated when *Trem2* is also depleted in 5× FAD;*CD33*^−/−^;*Trem2*^−/−^ mice, indicating that TREM2 functions downstream of CD33 in this pathway (Griciuc et al., 2019).

In addition to *APOE* and *CLU*, genetic variants in *ABCA7* have also been implicated in cholesterol metabolism pathways associated with AD risk. In addition to *TREM2*, common variants in *CD33*, *CR1*, and *MS4A* have been linked to dysregulation of the immune response, a central feature of AD pathogenesis. Furthermore, endocytosis-related genes such as *BIN1*, *PICALM*, *CD2AP*, *EPHA1*, and *SORL1* have been identified as harboring AD-associated genetic variants (Karch and Goate, 2015). The identification of these novel genetic risk factors has provided new opportunities to understand the underlying pathophysiology of AD, highlighting the role of key pathways involved in the disease process.

### Aging

Aging is the primary risk factor for sporadic AD. As organisms age, DNA damage accumulates, leading to an increase in the number of senescent cells. These cells then adopt a senescence-associated secretory phenotype (SASP), which releases proinflammatory cytokines, contributing to the development of age-related diseases (Guerrero et al., 2021).

The accumulation of somatic mutations in neurons during aging and the AD process is thought to be due to increased oxidative damage (Lodato et al., 2018; Miller et al., 2022). Furthermore, elevated levels of DNA double-strand breaks have been linked to structural variations in the genome and disruptions in 3D genome organization in excitatory neurons in the postmortem brains of AD patients (Dileep et al., 2023). Severe DNA damage has also been shown to induce senescence in various neurons, including Purkinje cells and cortical and hippocampal neurons, in aged mice (Jurk et al., 2012).

An increase in the number of senescent astrocytes has been observed in aging brains and in those with AD (Bhat et al., 2012; Turnquist et al., 2016). Similarly, dystrophic (senescent) microglia, which proliferate in aged and AD brains, have been associated with the early stages of tau pathology and neurodegeneration (Shahidehpour et al., 2021; Streit et al., 2009). Additionally, lipid droplet accumulation in microglia during aging has been documented in both mouse and human brains (Marschallinger et al., 2020).

Elimination of p16^INK4A^-positive senescent cells using the INK-ATTAC system has been shown to delay aging-associated disorders (Baker et al., 2011). Specifically, clearing senescent microglia and astrocytes in the brain using INK-ATTAC transgenic mice led to reductions in hyperphosphorylated tau, NFT formation and cognitive deficits in PS19 transgenic mice (Bussian et al., 2018). Senolytic treatment with dasatinib and quercetin selectively removes senescent oligodendrocyte progenitor cells (OPCs) from the plaque environment, resulting in decreased neuroinflammation and Aβ plaque deposition and improved cognitive function in APP/PS1 transgenic mice (Zhang et al., 2019). Moreover, the inhibition of microglial proliferation using the CSF1R inhibitor GW2580 has been shown to prevent the onset of senescence and reduce Aβ pathology in APP/PS1 transgenic mice (Hu et al., 2021).

Heterochronic parabiosis combined with intravenous injections of young mouse plasma has been shown to restore synaptic and neuronal protein levels, leading to improvements in working and associative memory in APP-transgenic mice, albeit without affecting amyloid plaque levels (Middeldorp et al., 2016). This approach was further explored in a phase 1 clinical trial (NCT02256306) (Sha et al., 2019). In a phase 2b/3 trial (NCT01561053), plasma exchange with albumin replacement in patients with mild-to-moderate AD resulted in improved memory, language abilities, processing speed, and quality of life (Boada et al., 2022).

### Environmental factors

In 2016, approximately 3.752 billion people (66.6% of the global population under the age of 49) were estimated to be infected with herpes simplex virus type 1 (HSV1) (James et al., 2020). Autopsy studies of AD specimens have detected HSV1 DNA in the brain (Jamieson et al., 1991). Moreover, HSV1 infection has been linked to an increased risk of AD, particularly in individuals carrying the *APOEε4* allele (Itzhaki et al., 1997; Linard et al., 2020; Lopatko Lindman et al., 2019). HSV1 infection dramatically accelerated Aβ plaque deposition both in 5× FAD mice and in a 3D human neural cell model (Cairns et al., 2020; Eimer et al., 2018). The use of anti-herpetic medications has been found to lower the risk of dementia in HSV-infected patients (Tzeng et al., 2018), and the administration of the herpes zoster vaccine has been demonstrated to protect against dementia (Eyting et al., 2023). Approximately 20%–60% of individuals with human immunodeficiency virus (HIV) infection experience cognitive impairment, known as HIV-associated neurocognitive disorder (HAND) (Nyamayaro et al., 2019; Wang et al., 2020b; Wei et al., 2020). The presence of amyloid plaques has been noted in the brains of individuals infected with HIV (Esiri et al., 1998). Moreover, human cytomegalovirus (CMV) infection has been associated with a 2.15-fold increased risk of developing AD (Barnes et al., 2015), and murine CMV infection has been shown to accelerate tau pathology in mouse fibroblasts and rat primary neurons (Mody et al., 2023).

Three specific bacterial species—*Borrelia burgdorferi* (Miklossy, 2016), *Chlamydia pneumoniae* (Balin et al., 1998; Gerard et al., 2006), and *Porphyromonas gingivalis* (Dominy et al., 2019)—have been implicated in the brains of AD patients. The presence of *Helicobacter pylori* in the gastric mucous membrane, serum, and plasma has also been observed to increase in individuals with AD (Kountouras et al., 2006; Malaguarnera et al., 2004). However, the role of infections in the pathogenesis of AD remains a topic of debate (Itzhaki et al., 2020).

Long-term exposure to air pollution has been linked to an increased risk of dementia, with heart failure and ischemic heart disease potentially amplifying the association between air pollution and dementia (Grande et al., 2020). Additionally, smoking has been associated with greater risks of both AD and vascular dementia than never smoking (Anstey et al., 2007; Rusanen et al., 2011).

### Lifestyle habits

Sleep disturbances have been linked to an increased risk of AD (Musiek and Ju, 2022). Extensive research indicates that conditions such as obstructive sleep apnea (OSA) and insufficient sleep duration are associated with a greater likelihood of cognitive impairment (Sabia et al., 2021; Yaffe et al., 2011). Notably, high sleep fragmentation, rather than short sleep duration, in adults aged 30–40 years, observed over a decade, is strongly associated with memory decline and cognitive impairment (Leng et al., 2024). Disturbed sleep quality and fragmented circadian rhythms are common in the years preceding AD, even before symptoms manifest (Ju et al., 2013; Musiek et al., 2018). Sleep deprivation hinders molecular clearance mechanisms in the brain (Eide et al., 2021), and disrupted sleep patterns, especially a lack of deep (slow-wave) sleep, are linked to elevated levels of Aβ and tau in the brain (Holth et al., 2019; Ju et al., 2017; Kang et al., 2009; Lucey et al., 2018). Sleep deprivation significantly increased Aβ deposition and tau pathology in APP/PS1 transgenic mice with the *APOEε4* allele but not in those with the *APOEε3* allele (Wang et al., 2023). Conversely, natural sleep or anesthesia significantly enhances the convective exchange of CSF with the ISF, promoting Aβ clearance (Xie et al., 2013).

Type 2 diabetes mellitus (T2DM), which is characterized by hyperglycemia, insulin resistance and peripheral inflammation, is implicated in increased AD risk (Profenno et al., 2010; Vagelatos and Eslick, 2013). T2DM is also associated with cerebrovascular disease and cognitive deficits (Messier et al., 2004; Ryan and Geckle, 2000). Aβ and hyperphosphorylated tau have been found in the pancreas of T2DM patients (Miklossy et al., 2010). Emerging evidence suggests that glucose hypometabolism, insulin resistance, and impaired insulin-like growth factor (IGF) signaling are linked to AD progression, leading to the characterization of AD as “type 3 diabetes” (Rivera et al., 2005; Steen et al., 2005).

High-fat diets (HFDs) are known to predispose individuals to obesity and diabetes by promoting insulin resistance (Morio et al., 2016). High intakes of saturated and trans-unsaturated fats have been associated with increased AD risk, while ω-6 polyunsaturated and monounsaturated fats appear to be protective (Morris et al., 2003). High caloric and fat intake levels are linked to an elevated AD risk, especially in individuals with the *APOEε4* allele (Luchsinger et al., 2002).

HFD-induced insulin resistance has been shown to promote Aβ generation, amyloid plaque deposition, and cognitive impairment in transgenic AD mice (Ho et al., 2004; Wakabayashi et al., 2019). However, one study revealed that HFD feeding induced microglial activation and cognitive deficits in both wild-type and 3× Tg AD mice without affecting Aβ or tau pathology (Knight et al., 2014). In contrast, early HFD feeding, before severe AD pathology, reduced Aβ plaque deposition and improved cognitive function in Tg6799 AD mice (Amelianchik et al., 2021).

High dietary sodium intake (≥12 g/d) has been associated with a 330% increased risk of cognitive impairment in older adults (Liu et al., 2023b). A high-salt diet (HSD) led to cerebral endothelial dysfunction and reduced cerebral blood flow through the gut-brain axis, resulting in cognitive impairment in mice (Faraco et al., 2018). Furthermore, HSD consumption induced tau hyperphosphorylation through the activation of calpain and CDK5, resulting in cognitive dysfunction in both wild-type and rTg4510 mice, although it did not affect Aβ levels in Tg2576 mice (Faraco et al., 2019).

Notably, a high dietary intake of vitamins C and E has been associated with a lower AD risk (Engelhart et al., 2002), possibly due to antioxidants reducing neuronal loss by protecting against oxidative damage (Christen, 2000).

### Cardiovascular and cerebrovascular disease

Cardiovascular disease (CVD) ranks as a leading cause of morbidity and mortality among older adults, underscoring its critical public health impact (Saeed et al., 2023). Increasing evidence connects the heightened risk of CVD, especially vascular dementia, with a greater likelihood of developing dementia. The key risk factors for CVD include high blood pressure, dyslipidemia, obesity, and diabetes, all of which have been well-documented in the literature (European et al., 2023; Livingston et al., 2017; Norton et al., 2014; Winblad et al., 2016). Notably, heart failure has been shown to increase AD risk by 1.8 times. Moreover, the use of antihypertensive medications appears promising for mitigating dementia risk. Additionally, a low diastolic pressure (below 70 mm Hg) further increases the risk of developing dementia (Qiu et al., 2006). Cerebral hypoperfusion is an early abnormality in both AD and vascular dementia patients, suggesting that it is a common abnormality. Midlife cardiovascular risk profiles have been associated with reduced cerebral perfusion later in life, highlighting the importance of cardiovascular health across the lifespan (Suri et al., 2019). Conversely, maintaining cardiovascular health is linked to reduced dementia risk and slower cognitive decline (Samieri et al., 2018).

Autopsy samples from up to 75% of individuals with AD reveal concurrent cerebral vascular pathology, indicating a significant overlap between AD and vascular conditions. Increased severity of cerebral atherosclerosis or arteriolosclerotic neuropathology is significantly associated with increased odds of AD (Arvanitakis et al., 2016). Furthermore, cerebral infarctions have been associated with an elevated risk of cognitive impairment and AD dementia, emphasizing the intertwined nature of cerebrovascular health and cognitive function (Schneider et al., 2004, 2007).

CAA is a prevalent cerebrovascular disease characterized by the deposition of Aβ in vessel walls (Yamada and Naiki, 2012). CAA frequently co-occurs with AD pathology in aging brains, accelerating the progression toward AD dementia. (Rabin et al., 2022).

Stroke, including both ischemic and hemorrhagic stroke, is the most prevalent form of cerebrovascular disease (Pan et al., 2020). In 2019, 12.2 million new cases of stroke were diagnosed, with a global prevalence of 101 million people living with this condition. Stroke has led to 6.55 million deaths worldwide, making it the second leading cause of death (GBD 2016 Stroke Collaborators, 2021). Approximately one in four adults is at risk of experiencing a stroke during their lifetime (GBD 2016 Lifetime Risk of Stroke 2018). The risk of developing dementia is notably greater in patients with severe stroke than in the general population, although this risk decreases significantly following milder strokes, such as transient ischemic attacks and minor strokes (McHutchison et al., 2019; Pendlebury et al., 2019). Ischemic stroke surgery has been shown to increase plasma and CSF tau levels, as well as ipsilateral cerebral tau pathology, in a transient middle cerebral artery occlusion (MCAO) mouse model (Laing et al., 2020).

Over 70% of individuals aged 50 and older show signs of at least one form of cerebral small vessel disease (CSVD), which can lead to acute stroke syndromes, MCI, and even dementia due to a chronic damage, small vessel blockage or leakage, blood‒brain barrier breakdown, and cerebral blood flow deficits (Hachinski et al., 2019; Wardlaw et al., 2019). Neurovascular dysfunction is known to exacerbate Aβ and tau pathology, further contributing to cognitive decline (Kisler et al., 2017). CSVD is a prevalent cause of vascular dementia and frequently coexists with AD pathology, highlighting the complex interplay between vascular and neurodegenerative processes in cognitive impairment (Wardlaw et al., 2019).

### Traumatic brain injury (TBI)

Substantial research has established a link between TBI and a heightened risk of developing AD at an earlier age (Fleminger et al., 2003; Gu et al., 2022; Nemetz et al., 1999; Weiner et al., 2014). Furthermore, postmortem examinations have revealed Aβ plaque accumulation in approximately one-third of TBI patients (Gentleman et al., 1997; Roberts et al., 1991; Smith et al., 2003). Studies on 5× FAD transgenic mice have shown that even mild TBI can precipitate and worsen BBB leakage, Aβ plaque formation, and cognitive impairments (Wu et al., 2021). Additionally, the levels of tau protein in CSF and peripheral blood increase following TBI, suggesting an acceleration of tau pathology (Olivera et al., 2015; Ost et al., 2006; Shahim et al., 2014). Moreover, repetitive mild TBI is associated with the development of chronic traumatic encephalopathy (CTE), a progressive tauopathy characterized by the absence of amyloid pathology (McKee et al., 2009).

### Other factors

High levels of inflammation have been associated with a 1.66-fold greater risk of dementia in individuals with metabolic syndrome (Yaffe et al., 2004). Additionally, chronic low-grade inflammation in the periphery has been connected to a 2.64-fold greater risk of AD in individuals possessing the *APOEε4* allele, particularly in the absence of CVD, leading to a 6.63-fold greater risk of AD (Tao et al., 2018). Notably, individuals who underwent systemic inflammatory challenges were more likely to experience cognitive impairment in subsequent years (Iwashyna et al., 2010).

Additionally, neuropsychiatric symptoms such as depression, aggression, anxiety, and sleep disorders are prevalent among AD patients (Zhao et al., 2016a). Notably, depression and anxiety have been identified as significant predictors of an increased risk for AD, with a 2.13-fold increase in depression and a 1.53-fold increase in anxiety (Becker et al., 2018; Green et al., 2003).

A lack of social engagement has been linked to an increased risk of developing dementia (Penninkilampi et al., 2018), whereas strong social ties have been shown to reduce the risk of dementia by 30%–50% (Sommerlad et al., 2023). Furthermore, risk factors such as heavy alcohol use (Rehm et al., 2019) and hearing loss (Thomson et al., 2017) have been identified as contributing to an increased risk of dementia. On a more positive note, higher levels of education have consistently demonstrated a protective effect against the development of AD (McDowell et al., 2007; Sando et al., 2008), suggesting that cognitive reserve may play a role in mitigating risk.

## Biomarkers

In 2018, the National Institute on Aging and Alzheimer’s Association (NIA-AA) introduced the ATN biomarker framework (as described previously), which provides guidelines for categorizing biomarkers and classifying AD patients according to their biomarker profiles (Jack et al., 2018) (Table 1). Despite its introduction, there remains a discussion regarding its efficacy, given that it does not entirely capture the multifaceted nature of AD pathophysiology.

**Table 1.** Biomarkers in Alzheimer’s disease.

Category	Biomarker	Change in AD	Disease stage	Additional notes
**Imaging**	MRI	Reduced volume in hippocampus and entorhinal cortex	Preclinical and early AD	Sensitive to early changes, but not specific to AD
	^18^F-FDG PET	Decreased glucose metabolism in affected areas	Early and later AD	Reflects neuronal dysfunction, but not specific to AD
	Aβ PET	Increased amyloid plaques	Preclinical and early AD	High specificity for AD pathology
	Tau PET	Increased tau tangles	Early and later AD	Emerging technology with promising results, but still under development
**CSF**	Aβ_42_/Aβ_40_	Decreased	Preclinical and early AD	Early detection
	Total tau	Increased	Early and later AD	Reflects neuronal damage and tau pathology, but not specific to AD
	p-Tau	Increased, particularly pTau217, pTau181, and pTau231	Preclinical and later AD	More specific to AD than total tau, pTau217 performs best
	NfL	Increased	Later AD	Reflects axonal damage and neurodegeneration, but not specific to AD
	GFAP	Increased	Early AD	Reflects astrocyte activation and neuroinflammation
**Blood**	Aβ_42_/Aβ_40_	Potentially decreased, but research is ongoing	Preclinical and early AD	Non-invasive and accessible, but results not yet definitive
	Total tau	Increased	Early and later AD	Reflects neuronal damage and tau pathology, but not specific to AD
	Brain-derived tau	Increased	Early and later AD	Specific to AD
	p-Tau	Increased, particularly pTau217, pTau181, and pTau231	Preclinical and later AD	Specific to AD, pTau217 performs best, still under development
	NfL	Increased	Later AD	Reflects axonal damage and neurodegeneration, but not specific to AD
	GFAP	Increased	Early AD	Reflects astrocyte activation and neuroinflammation

### Imaging biomarkers

MRI is recommended for assessing cognitive impairment, as it aids in excluding other potential causes and evaluating brain atrophy (Likeman et al., 2005; Zivanovic et al., 2023). Elevated levels of Aβ, identified through PET scans, are linked to increased atrophy in specific brain regions, such as the temporal and parietal lobes, which is associated with cognitive decline (Knopman et al., 2016). While hippocampal atrophy detected by MRI is a marker of AD, it can also be detected in various other conditions, including cerebrovascular disease, FTD, and hippocampal sclerosis (Chiang et al., 2010).

Aβ PET imaging plays a crucial role in excluding AD by tracking Aβ accumulation and facilitating early detection in the disease’s initial stages (Grothe et al., 2017a). ^18^F-FDG PET is valuable for diagnosing neurodegenerative diseases and forecasting short-term outcomes, with the extent of hypometabolism observed correlating with cognitive decline severity, thus serving as an important marker of AD progression (Iaccarino et al., 2019; Rhodius-Meester et al., 2020). Tau PET imaging allows for the detection of tau pathology, although it is not exclusive to AD (Ossenkoppele et al., 2018). Unlike Aβ PET, tau PET imaging patterns are strongly correlated with cognitive function and clinical AD phenotypes (Aschenbrenner et al., 2018; Ossenkoppele et al., 2016), and tau PET abnormalities are closely aligned with ^18^F-FDG PET hypometabolism (Ossenkoppele et al., 2016).

However, the use of PET tracers is limited by their high costs and the need for specialized infrastructure (Ashton et al., 2021). To overcome these limitations, fluid-based biomarkers have been developed that offer increased sensitivity and convenience for detecting AD-related pathologies.

### Fluid biomarkers

Compared to Aβ_40_, Aβ_42_ monomers are more prone to forming insoluble plaques, resulting in a decrease in the extracellular Aβ_42_ concentration and the Aβ_42_/Aβ_40_ ratio (Lewczuk et al., 2017; Roher et al., 1993). A lower Aβ_42_/Aβ_40_ ratio in CSF and plasma is associated with Aβ PET findings, allowing for the specific monitoring of disease progression by measuring Aβ_42_ and Aβ_40_ levels (Lewczuk et al., 2017; Li et al., 2022).

Owing to its soluble nature and multiple PTMs in CSF and plasma, tau protein is recognized as a sensitive and robust biomarker for tau pathology and neurodegeneration in AD (Self and Holtzman, 2023). Compared to total tau, plasma brain-derived tau (BD-tau) shows a better ability to distinguish AD from other neurodegenerative diseases (Gonzalez-Ortiz et al., 2023). Increases in CSF levels of phosphorylated tau at T181, T217, and T231 in early preclinical AD stages can accurately differentiate between Aβ PET-positive and Aβ PET-negative individuals (Suarez-Calvet et al., 2020). pTau181, which is closely correlated with total tau levels in CSF, is markedly elevated in AD but not in most other neurodegenerative conditions, making it the gold standard for assessing phosphorylated tau levels and an AD-specific marker (Skillback et al., 2015). Among these, plasma pTau217 shows the most promise in distinguishing patients with MCI who have abnormal brain Aβ levels or are likely to progress to AD dementia (Ashton et al., 2024; Barthelemy et al., 2024; Janelidze et al., 2023; Palmqvist et al., 2020). Notably, plasma pTau217 exhibits clinical performance comparable to or even better than that of currently FDA-approved CSF-based tests for classifying Aβ and tau PET status (Barthelemy et al., 2024).

Although neurofilament light chain (NfL), a marker of neuroaxonal degeneration, may not serve as an ideal standalone diagnostic marker for AD (Benkert et al., 2022; Karantali et al., 2022; Mattsson et al., 2017; van der Ende et al., 2022), it is useful for monitoring and predicting disease severity (Jung and Damoiseaux, 2024). Elevated NfL levels in the blood or CSF correlate with reduced cognitive function, advanced neurodegeneration, and rapid cognitive decline in patients with AD (Mattsson et al., 2019; Moscoso et al., 2021; Sugarman et al., 2020; Weston et al., 2017). Additionally, lower levels of neuronal pentraxin-2 (NPTX2) and higher levels of synaptosomal-associated protein 25 (SNAP25) in CSF are linked to AD progression (Galasko et al., 2019). Blood levels of glial fibrillary acidic protein (GFAP), a marker for neuroinflammation, have shown potential in differentiating AD from FTD and predicting the progression from MCI to AD dementia (Cicognola et al., 2021; Oeckl et al., 2022).

CSF-based biomarkers, while scalable and cost-effective, allowing for the evaluation of multiple markers from a single sample, lack pathological localization and require invasive procedures such as lumbar puncture (Leuzy et al., 2021). Blood-based biomarkers offer a more accessible and scalable option, although they also lack pathological localization (Teunissen et al., 2022). However, challenges remain in identifying effective biomarkers, validating them in real-world populations, and developing diagnostic assays for clinical use (Teunissen et al., 2022).

Enhancing the depth and precision of biomarker characterization across the AD continuum is necessary. Such advancements will support the development of targeted therapeutic approaches in the future.

## Prevention strategies

Pharmacological treatments for AD are limited to symptom relief, with three cholinesterase inhibitors (donepezil, rivastigmine, and galantamine) and the NMDA receptor antagonist memantine. These drugs can improve cognitive function and daily living activities but do not halt disease progression (Knopman et al., 2021).

To date, 187 clinical trials have tested 141 treatments for AD across various phases. These trials have focused on diverse mechanisms, including Aβ (16%), neurotransmitters (29%), inflammation (17%), synaptic plasticity/neuroprotection (13%), and tau (9%) (Cummings et al., 2023).

Recent advancements in clinical trials with anti-Aβ antibodies, such as donanemab, lecanemab, and aducanumab, have shown promise in reducing Aβ levels and decelerating cognitive decline (Budd Haeberlein et al., 2022; Sims et al., 2023; van Dyck et al., 2023). Notably, compared with the placebo, lecanemab (leqembi), a humanized IgG1 monoclonal antibody targeting soluble Aβ protofibrils, has been shown to delay cognitive decline by 5.3 months over 18 months, leading to its traditional approval by the FDA in 2023 for AD treatment (van Dyck et al., 2023). The success of Aβ-targeting antibodies in early AD therapy offers the first clinicopathological indication that a disease-modifying treatment for AD is feasible (Jucker and Walker, 2023). Despite showing promise in clearing amyloid from the brain, the clinical benefits of anti-Aβ antibodies are limited, and safety concerns, including amyloid-related imaging abnormalities (ARIAs), necessitate a deeper understanding of the underlying mechanisms of AD to refine treatments.

Patients undergoing immunotherapy may experience significant side effects, such as ARIAs, including edema/effusion (ARIA-E) and microhemorrhages (ARIA-H), which are particularly linked to CAA and the presence of the *APOEε4* allele (Greenberg et al., 2020; Salloway et al., 2022; Sims et al., 2023; Sveikata et al., 2022; van Dyck et al., 2023; Villain et al., 2022). Early diagnosis through precise biomarkers is crucial for minimizing these risks and enhancing treatment efficacy (Jucker and Walker, 2023).

Given the challenge of transporting antibodies across the BBB, with less than 0.1% of circulating antibodies penetrating it (Poduslo et al., 1994), high doses are needed. Techniques such as ultrasound to temporarily open the barrier may facilitate amyloid removal and reduce necessary antibody dosages, despite potential adverse effects (Rezai et al., 2024).

In addition to passive Aβ immunization, five Aβ-targeting vaccine candidates are currently undergoing clinical trials: ACI-24, ABvac40, UB-311, AV-1959D, and ALZ-101 (Wu et al., 2024).

Considering the stronger correlation between tau pathologies and the severity of dementia compared to amyloid plaques, targeting tau has emerged as a potentially more effective strategy for combating AD. Among the most promising avenues in this regard is tau immunotherapy, which encompasses both active vaccines, such as AADvac1 and ACI-35, and a range of antibodies, including APNmAb005, E2814, JNJ-63733657, Lu AF87908, MK-2214, PNT001, PRX005, semorinemab, and bepranemab. These therapies are currently being evaluated in clinical trials (Congdon et al., 2023). Notably, E2814, a humanized monoclonal IgG1 antibody targeting the HVPGG epitope in the microtubule-binding domain of tau, has demonstrated potential in reducing tau seeding, spreading, and intracellular deposition in tau-P301S transgenic mice (Roberts et al., 2020) and is now in a phase III trial (NCT01760005). Moreover, the tau-targeting antisense oligonucleotide (ASO) MAPTRx has shown safety and a sustained reduction in CSF tau levels in a first-in-human phase 1b trial for patients with mild AD (NCT03186989) (Mummery et al., 2023). However, TRx0237 (LMTX), a second-generation tau protein aggregation inhibitor, did not demonstrate benefits for patients with mild-to-moderate AD or FTD in phase 3 trials (Gauthier et al., 2016; Shiells et al., 2020).

In parallel, targeting the innate immune system has shown promise. Repeated administration of a TREM2 agonist antibody (AL002) increased plaque-associated microglia and reduced amyloid deposition in 5× FAD mice, including those expressing both the common variant and the R47H variant of human TREM2 (Wang et al., 2020a), and has progressed to phase 2 trials for early AD (NCT04592874). Furthermore, masitinib, an orally administered tyrosine kinase inhibitor targeting mast cells and microglia, has shown efficacy in a phase 3 trial (NCT01872598) by significantly reducing cognitive decline in patients with mild-to-moderate AD (Dubois et al., 2023).

Additionally, 40 Hz gamma-sensory stimulation has been investigated as a novel therapeutic approach, with studies demonstrating its potential to enhance glymphatic clearance of Aβ and reduce the microglial inflammatory response, synapse loss, amyloid plaque deposition, and tau pathology, thereby restoring cognitive function in AD mice (Adaikkan et al., 2019; Iaccarino et al., 2016; Martorell et al., 2019; Murdock et al., 2024). However, contrasting findings were reported in another study in which neither amyloid plaque nor microglial morphology was affected by 40 Hz stimulation in AD mice (Soula et al., 2023). A recent phase 2 trial exploring combined visual and auditory 40 Hz gamma-sensory stimulation over six months (NCT03556280) indicated reduced total and regional white matter atrophy and myelin content loss in participants receiving active treatment compared to those receiving sham treatment (Da et al., 2024). These findings underscore the diverse and innovative strategies being explored to address the complex pathology of AD, with a shift toward more targeted and potentially disease-modifying therapies.

## Conclusion

AD remains a challenging and complex neurodegenerative condition, and significant advancements in understanding its pathology have been achieved over the last three decades. Despite extensive research efforts aimed at identifying key proteins such as Aβ and tau and strategies to mitigate neuroinflammation, achieving breakthrough clinical outcomes has been challenging. These challenges stem from a variety of factors, including the properties of the drugs, the timing of treatment, and the inherent variability of the disease itself.

**Figure 4.**
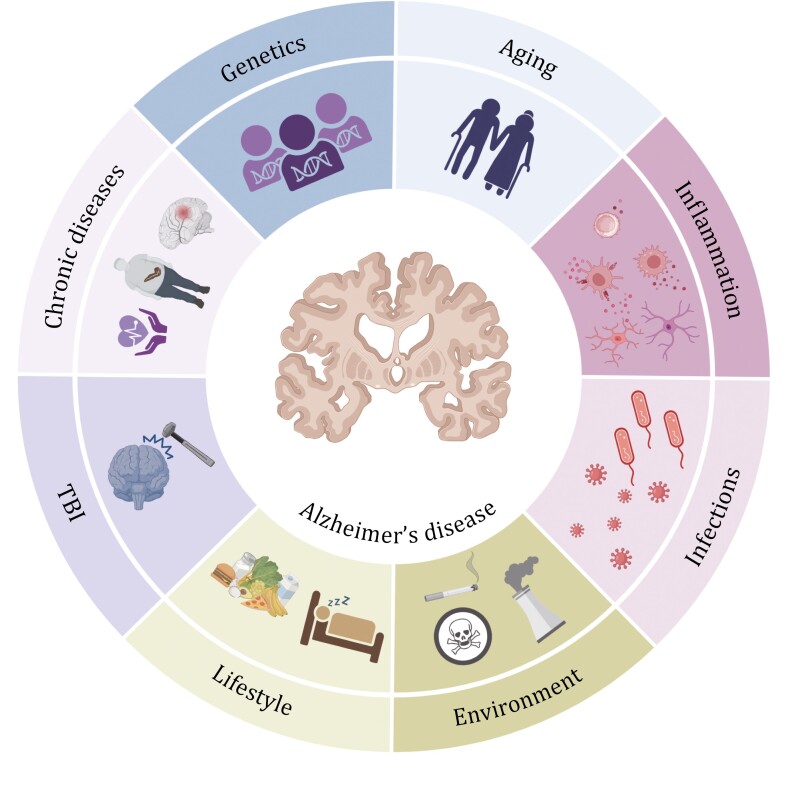
Diverse risk factors contributing to AD pathogenesis. AD is a complex neurodegenerative disease influenced by a multitude of risk factors. These include genetic predisposition, the natural aging process, systemic inflammation, the presence of chronic diseases (type 2 diabetes, cardiovascular and cerebrovascular diseases), infections, traumatic brain injury (TBI), lifestyle choices (sleep patterns, high-fat, and high-salt diets), and environmental exposures. Additional factors that may affect AD incidence include neuropsychiatric symptoms, social engagement, alcohol consumption, hearing impairment, and educational attainment. The intricate interactions among these factors lead to the progressive neurodegeneration characteristic of AD.

The pathophysiology of AD is intricate and influenced by a combination of genetic predispositions, the natural aging process, systemic inflammation, chronic diseases, infections, TBI, lifestyle choices, and environmental factors (Fig. 4). These elements collectively drive the progressive neurodegeneration that spans from the initial amyloid pathology to the manifestation of overt dementia over a period of 15–20 years. Certain modifiable risk factors may further accelerate the progression of the disease, highlighting the importance of ongoing, intensive research to uncover new therapeutic strategies.

The critical role of early intervention in the effective management of AD has become increasingly apparent, underscoring the need for the identification of sensitive and specific biomarkers. Biomarkers detectable in easily accessible biofluids, such as blood, are particularly crucial for early diagnosis, disease monitoring, and personalized treatment approaches. Although the recent FDA approval of anti-Aβ monoclonal antibodies represents a step forward, their limited clinical efficacy highlights the necessity for more targeted pharmacological interventions. These interventions should be informed by biomarkers, moving beyond the traditional one-size-fits-all treatment approach. The adoption of biomarker-guided therapies that target specific molecular pathways offers a promising avenue for improving treatment outcomes and ultimately reducing the burden of AD.

## Data Availability

Not applicable.
